# *Orientia tsutsugamushi* uses two Ank effectors to modulate NF-κB p65 nuclear transport and inhibit NF-κB transcriptional activation

**DOI:** 10.1371/journal.ppat.1007023

**Published:** 2018-05-07

**Authors:** Sean M. Evans, Kyle G. Rodino, Haley E. Adcox, Jason A. Carlyon

**Affiliations:** Department of Microbiology and Immunology, Virginia Commonwealth University Medical Center, School of Medicine, Richmond, Virginia, United States of America; Purdue University, UNITED STATES

## Abstract

*Orientia tsutsugamushi* causes scrub typhus, a potentially fatal infection that threatens over one billion people. Nuclear translocation of the transcription factor, NF-κB, is the central initiating cellular event in the antimicrobial response. Here, we report that NF-κB p65 nuclear accumulation and NF-κB-dependent transcription are inhibited in *O*. *tsutsugamushi* infected HeLa cells and/or primary macrophages, even in the presence of TNFα. The bacterium modulates p65 subcellular localization by neither degrading it nor inhibiting IκBα degradation. Rather, it exploits host exportin 1 to mediate p65 nuclear export, as this phenomenon is leptomycin B-sensitive. *O*. *tsutsugamushi* antagonizes NF-κB-activated transcription even when exportin 1 is inhibited and NF-κB consequently remains in the nucleus. Two ankyrin repeat-containing effectors (Anks), Ank1 and Ank6, each of which possess a C-terminal F-box and exhibit 58.5% amino acid identity, are linked to the pathogen’s ability to modulate NF-κB. When ectopically expressed, both translocate to the nucleus, abrogate NF-κB-activated transcription in an exportin 1-independent manner, and pronouncedly reduce TNFα-induced p65 nuclear levels by exportin 1-dependent means. Flag-tagged Ank 1 and Ank6 co-immunoprecipitate p65 and exportin 1. Both also bind importin β1, a host protein that is essential for the classical nuclear import pathway. Importazole, which blocks importin β1 activity, abrogates Ank1 and Ank6 nuclear translocation. The Ank1 and Ank6 regions that bind importin β1 also mediate their transport into the nucleus. Yet, these regions are distinct from those that bind p65/exportin 1. The Ank1 and Ank6 F-box and the region that lies between it and the ankyrin repeat domain are essential for blocking p65 nuclear accumulation. These data reveal a novel mechanism by which *O*. *tsutsugamushi* modulates the activity and nuclear transport of NF-κB p65 and identify the first microbial proteins that co-opt both importin β1 and exportin 1 to antagonize a critical arm of the antimicrobial response.

## Author summary

*Orientia tsutsugamushi* is a genetically intractable obligate intracellular bacterial pathogen that invades professional phagocytes and other host cells to cause scrub typhus, a potentially deadly human disease that significantly impacts global health. How *O*. *tsutsugamushi* modulates immune signaling is poorly understood. In this study, we demonstrate that *O*. *tsutsugamushi* inhibits the accumulation of the NF-κB subunit, p65, in the nucleus and NF-κB mediated transcription, both of which are critical for the antimicrobial response. We link these phenomena to two *O*. *tsutsugamushi* effectors that bind importin β1 and exploit its nuclear import function to enter the nucleus, inhibit NF-κB-dependent transcriptional activation, interact with exportin 1 and p65, and co-opt exportin 1 activity to mediate p65 nuclear export. We evidence the first examples of microbial proteins that exploit both importin and exportin activities to modulate NF-κB nuclear transport. As NF-κB is also associated with inflammation, autoimmunity, and cancer, dissecting how microbial proteins modulate NF-κB could not only lead to better understanding of infectious disease processes, but also could yield information that can ultimately be exploited to treat disorders caused by NF-κB dysregulation.

## Introduction

Scrub typhus is a neglected zoonosis long known to be endemic to the Asia-Pacific, where one billion people are at risk and one million cases are reported annually [1]. Hundreds of American and Allied soldiers died from and several thousand became ill with scrub typhus during World War II [2–5]. Scrub typhus was the second leading cause of febrile illness among troops during the Korean War and Vietnam conflict [2, 4, 6–8]. Confirmed cases and serologic evidence in Middle Eastern, African, and South American countries indicate that the geographic range of and at-risk population for the disease are likely much larger [9–13]. Symptoms include fever, maculopapular rash, pneumonitis, and meningitis, with progression to disseminated intravascular coagulation, circulatory collapse, and organ failure [1, 2, 14, 15]. If left untreated or antibiotic therapy delayed, scrub typhus carries a median mortality rate of 6%, but can be as high as 70% [1].

The etiologic agent of scrub typhus is *Orientia tsutsugamushi*, a genetically intractable obligate intracellular bacterium that is vectored by the larval (chigger) stage of trombiculid mites [1]. *O*. *tsutsugamushi* invades dermal leukocytes at the chigger bite site and spreads systemically to multiple organs where it proliferates in leukocytes and endothelial cells to cause organ-specific inflammation [16, 17]. *O*. *tsutsugamushi* infected macrophages have been detected in the lymph node, liver, and spleen of scrub typhus patients and in the peritoneal cavity, spleen, and lungs of experimentally infected mice [16, 18–20]. In a recent mouse model study that approximated natural infection, the *O*. *tsutsugamushi* load was greatest in the lungs where it predominantly infected macrophages [16]. Thus, macrophages are important *in vivo* target host cells for *O*. *tsutsugamushi*.

The ability of *O*. *tsutsugamushi* to replicate in the cytosol of diverse host cell types, including professional phagocytes, suggests that it likely evolved to modulate processes that occur in response to intracellular infection. Yet, despite the pathogen’s impact on global health, whether and how it manipulates the antimicrobial response are poorly understood. Of 1,912 bacterial genomes examined, the *O*. *tsutsugamushi* Ikeda strain ranks in the top 0.3% in terms of the number of genes encoding ankyrin repeat-containing proteins (Anks) [21]. Most *O*. *tsutsugamushi* Anks also carry a C-terminal F-box motif that is capable of binding SKP1 of the SCF1 ubiquitin ligase complex [22, 23]. Evidence for the importance of these Type 1 secretion system effectors to *O*. *tsutsugamushi* pathobiology continues to mount, as the bacterium transcriptionally expresses its entire Ank repertoire during infection of mammalian cells and select Anks have been shown to co-opt or modulate SCF1 ubiquitin ligase assembly, Golgi-to-endoplasmic reticulum (ER) retrograde trafficking, ER stress, ER-associated degradation, and protein secretion [22–26].

Activation of the transcription factor, nuclear factor kappa-light-chain-enhancer of activated B cells (NF-κB) is the central initiating cellular event in the antimicrobial response (reviewed in [27]). The mammalian NF-κB family consists of five proteins that function by forming homo- or heterodimers [28]. Of these, ubiquitously expressed p50/p65 is the most studied and well understood. p50/p65 is held inactive in the cytosol by binding of NF-κB inhibitor α (IκBα) to the nuclear localization signal (NLS) of p65. Signaling cascades initiated by stimuli, such as exposure to pathogen-associated molecular patterns or TNFα, activate the IκB kinase (IKK) complex. Next, IKKβ phosphorylates IκBα, targeting IκBα for ubiquitination and proteasomal degradation. With the p65 NLS no longer occluded by IκBα, p50/p65 translocates into the nucleus where it binds to DNA κB sites to activate hundreds of antimicrobial and pro-inflammatory genes [27].

The NF-κB response during *O*. *tsutsugamushi* infection is poorly understood. An electrophoretic mobility shift assay indicated NF-κB activation in *O*. *tsutsugamushi* infected J774.1 macrophage-like cells at two h [29]. Other time points were not examined. A similar study performed using immortalized human microvascular endothelial cells evidenced NF-κB activation during the first four h of infection but not at eight h [30], suggesting that the bacterium might modulate NF-κB. Despite these interesting preliminary leads made between 2000 and 2002, questions as to whether *O*. *tsutsugamushi* inhibits NF-κB, if it does so in a primary target host cell, and the responsible bacterial factors have since remained unanswered. Herein, we confirm that *O*. *tsutsugamushi* reduces NF-κB accumulation in the nucleus and inhibits NF-κB-dependent gene expression. Its ability to do so is linked to two of its T1SS effectors, Ank1 and Ank6, which phenocopy p65 inhibition observed during infection and do so by not only binding p65 but also by exploiting importin β1 and exportin 1, host proteins that are essential for transporting cargo into and out of the nucleus, respectively [31, 32]. We reveal a complex mechanism by which *O*. *tsutsugamushi* inhibits the NF-κB response.

## Results

### *O*. *tsutsugamushi* inhibits p65 accumulation in the nucleus

**Fig 1:**
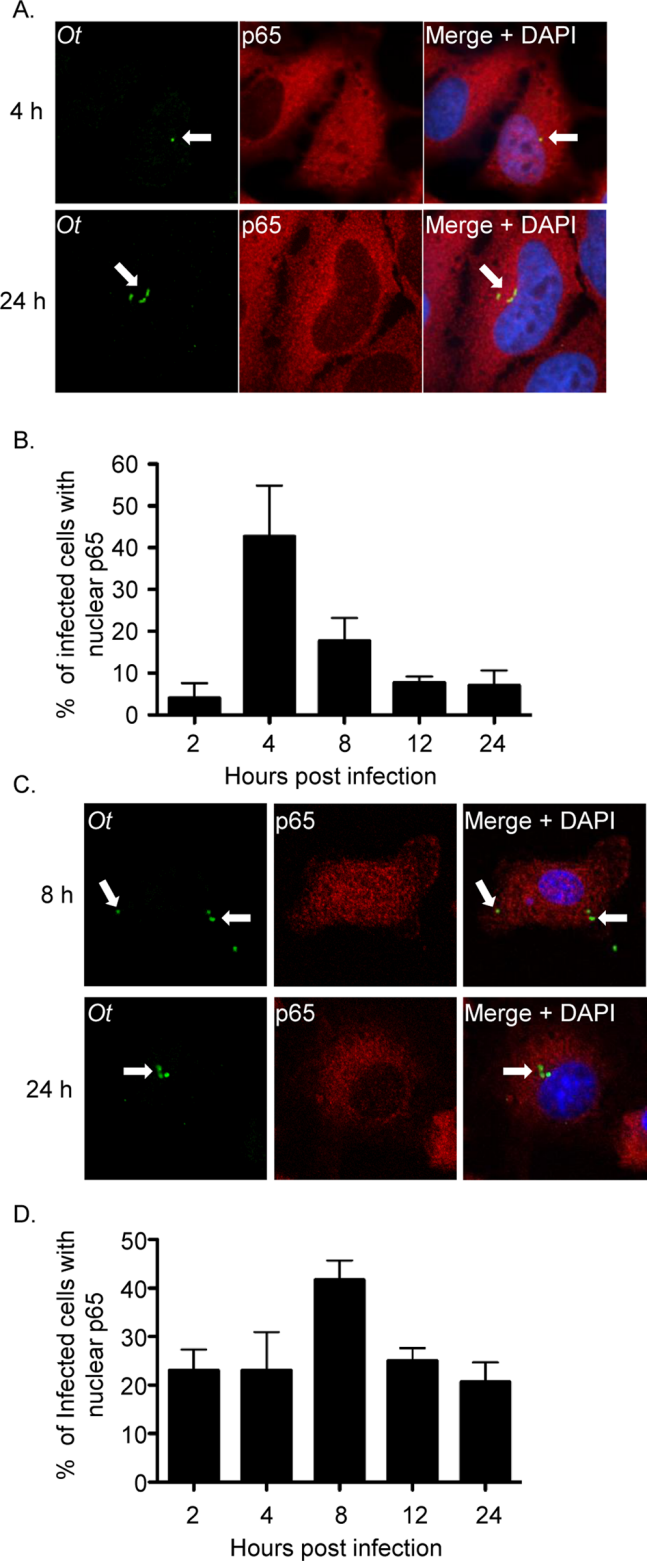
*O*. *tsutsugamushi* inhibits p65 nuclear accumulation. HeLa cells (A and B) or BMDMs (C and D) were infected with *O*. *tsutsugamushi* at an MOI of 1. At 2, 4, 8, 12, or 24 h, the cells were fixed and screened with antibodies against *O*. *tsutsugamushi* TSA56 (*Ot*) and p65 prior to examination by confocal microscopy. (A and C) Representative fluorescence images of cells viewed for *Ot*, p65, and merged images plus DAPI, which stains the nucleus, are presented. White arrows denote representative individual *O*. *tsutsugamushi* bacteria. (B and D) The mean percentage + SD of cells exhibiting p65 in the nucleus was determined at each time point. Triplicate samples of 100 cells each were counted per time point. Results are representative of three separate experiments.

For NF-κB to activate antimicrobial gene transcription, it must first translocate into the nucleus [27]. To determine if NF-κB accumulation in the nucleus is altered over the course of *O*. *tsutsugamushi* infection, HeLa cells, which are useful for studying *O*. *tsutsugamushi* cellular microbiology [22, 33], or murine bone marrow-derived macrophages (BMDMs) were infected and examined by confocal microscopy. In infected HeLa cells, p65 was detected in 42.7% of nuclei by 4 h, but only 17.7% by 8 h, 7.7% at 12 h, and 7.0% at 24 h (Fig 1A and 1B). A similar trend was observed for infected BMDMs, except that the percentage of infected cells in which nuclear p65 was detectable peaked at 8 h and the reduction observed at 12 and 24 h was not as pronounced as that for HeLa cells (Fig 1C and 1D). p65 was absent from the nuclei of both host cell types harboring as few as three bacteria (Fig 1A and 1C). These data suggest that *O*. *tsutsugamushi* begins to inhibit p65 accumulation in the nucleus within the first several hours following host cell invasion.

**Fig 2:**
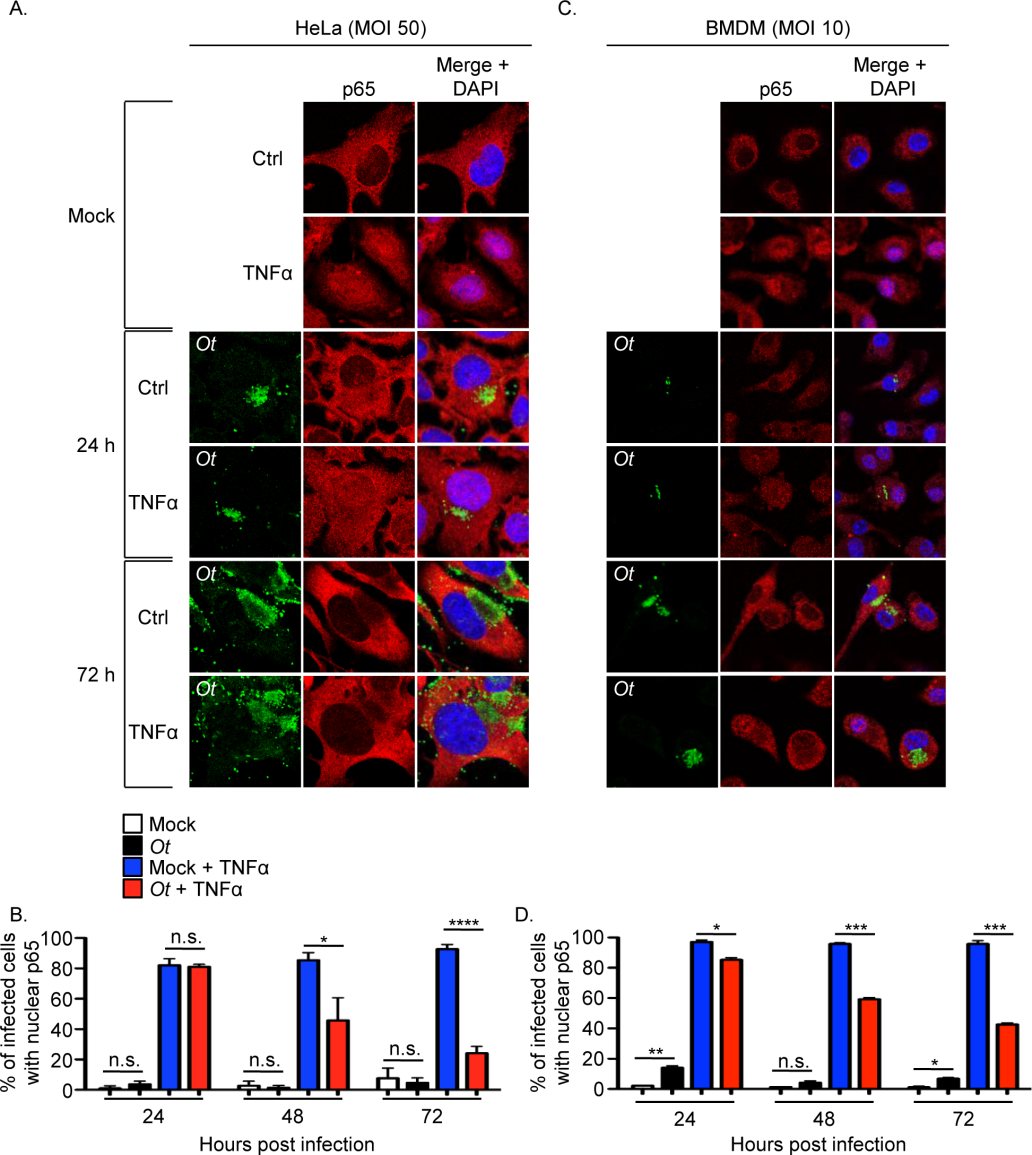
*O*. *tsutsugamushi* inhibits TNFα-stimulated p65 nuclear accumulation. HeLa cells (A and B) or BMDMs (C and D) were infected with *O*. *tsutsugamushi* at an MOI of 50 or 10, respectively, or mock infected. At 24, 48, or 72 h, the cells were treated with TNFα or vehicle control (Ctrl) for 30 min after which they were fixed, screened with antibodies against *O*. *tsutsugamushi* TSA56 (*Ot*) and p65, and visualized by confocal microscopy. (A and C) Representative fluorescence images of cells viewed for *Ot*, p65, and merged images plus DAPI, which stains the nucleus, are presented. (B and D) The mean percentage + SD of cells exhibiting p65 in the nucleus were determined at each time point. Triplicate samples of 100 cells each were counted per time point. Statistically significant (*P < 0.05; **P<0.01; ***P < 0.001; ****P < 0.0001) values are indicated. n.s., not significant. Data are the mean + SD of three independent experiments performed in triplicate.

TNFα, a cytokine that is present in the sera of scrub typhus patients and mice experimentally infected with *O*. *tsutsugamushi*, activates NF-κB [27, 34–38]. Therefore, it was examined if the bacterium is capable of inhibiting p65 accumulation in the nucleus following host cell exposure to TNFα. For these experiments, we selected multiplicities of infection (MOIs) that were reflective of what has been observed in leukocytes in the eschars of scrub typhus patients [17]. HeLa cells and BMDMs were incubated with *O*. *tsutsugamushi* organisms at MOIs of 50 and 10, respectively, for 24, 48, or 72 h followed by incubation in the presence of TNFα or vehicle control for 30 min. Importantly, both cell types remain intact at 72 h even following inoculation with *O*. *tsutsugamushi* at MOIs as high as 100 (S1 and S2 Figs). In the absence of TNFα, p65 was detected in the nuclei of only a small percentage of infected cells, similar to mock infected controls (Fig 2), indicating that the lack of a robust NF-κB response observed during the first 24 h continues at 48 and 72 h. p65 pronouncedly accumulated in the nuclei of mock infected cells exposed to TNFα. In contrast, *O*. *tsutsugamushi* infection strongly reduced the percentages of TNFα-stimulated HeLa cells and BMDMs in which p65 could be detected in the nucleus beginning at 48 h. The robustness of this inhibition increased over the duration of infection. Thus, *O*. *tsutsugamushi* inhibits TNFα-stimulated p65 accumulation in the nucleus and its ability to do so increases as infection proceeds.

### p65 levels are not reduced in *O*. *tsutsugamushi* infected cells

**Fig 3:**
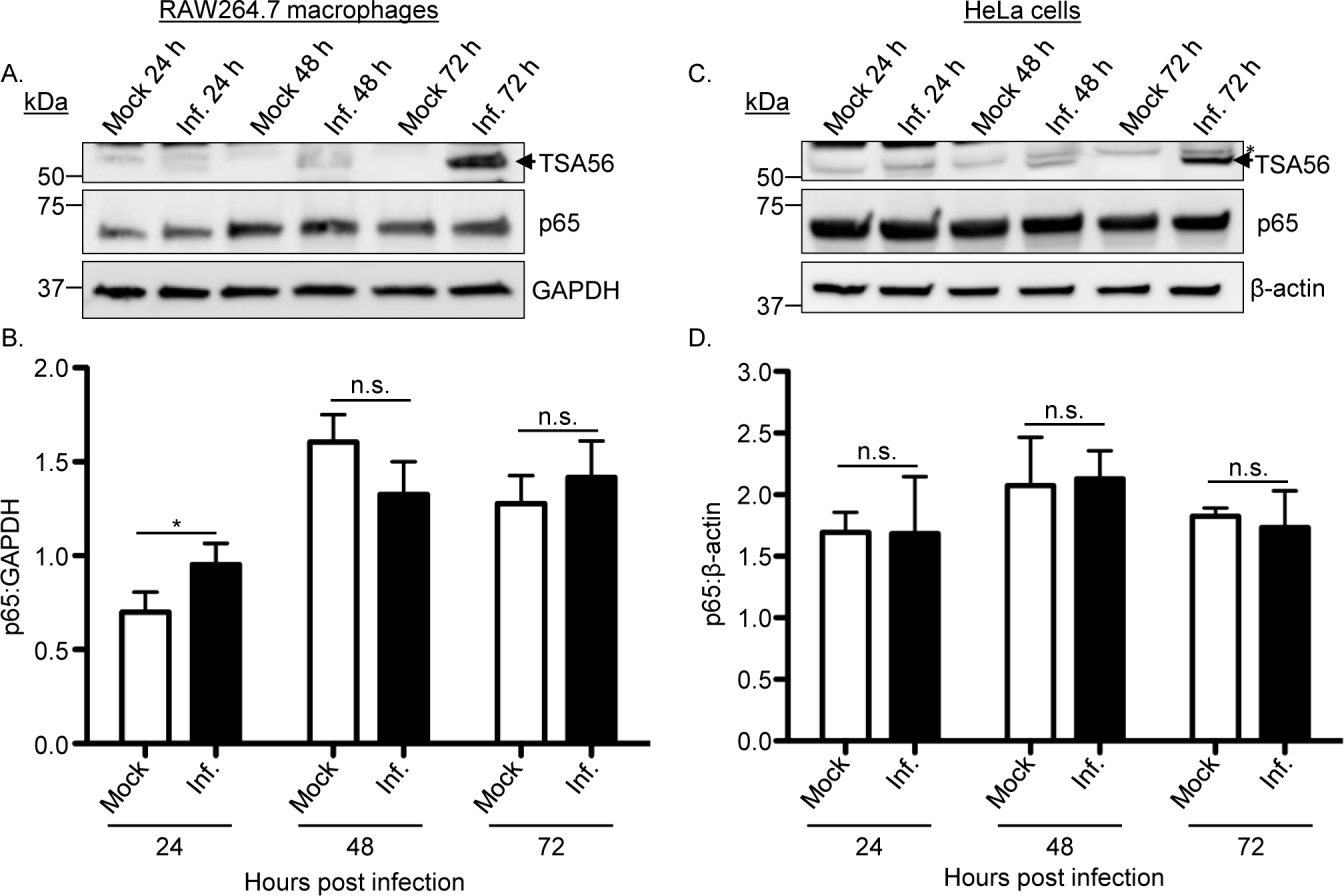
p65 levels are unchanged in *O*. *tsutsugamushi* infected cells. RAW264.7 (A and B) or HeLa cells (C and D) were infected with *O*. *tsutsugamushi* at an MOI of 25 or 10, respectively, or mock infected. (A) Whole cell lysates were analyzed by Western blotting with antibodies to *O*. *tsutsugamushi* TSA56 and p65. The blots were stripped and reprobed with antibody against GAPDH or β-actin as a loading control. (B and D) Mean normalized ratios + SD of p65:GAPDH (B) and p65:β-actin (D) from three separate experiments were calculated using densitometry. Statistically significant (*P < 0.05) values are indicated. n.s., not significant. The arrowhead and asterisk in A and B denote the expected size for TSA56 and a non-specific host cell-derived band, respectively.

The reduction in p65 accumulation in the nuclei of *O*. *tsutsugamushi* infected cells following TNFα stimulation could be due to the bacterium promoting degradation of p65, preventing degradation of IκBα as a means of keeping p65 sequestered in the cytosol in its inactive state, or directly acting on p65 itself. To determine if the reduction of p65 in the nuclei of *O*. *tsutsugamushi* infected cells was due to it being degraded, RAW264.7 macrophages and HeLa cells were incubated with the bacteria at MOIs of 25 and 10, respectively. Whole cell lysates recovered at 24, 48, and 72 h were subjected to Western blot and densitometry analyses. Probing with GAPDH and β-actin antibodies confirmed that equal protein amounts per sample had been loaded (Fig 3A and 3C). Antibody against the *O*. *tsutsugamushi* immunodominant protein, TSA56 (56-kDa type-specific antigen) [24, 39], verified that the appropriate samples were infected and that the bacterial load increased over the course of infection. p65 levels were not reduced in *O*. *tsutsugamushi* infected versus control cells at all time points for both host cell types (Fig 3). These data demonstrate that regardless of the bacterial load or duration of infection *O*. *tsutsugamushi* does not reduce cellular levels of p65.

### *O*. *tsutsugamushi* modulates p65 nuclear accumulation without inhibiting IκBα degradation

**Fig 4:**
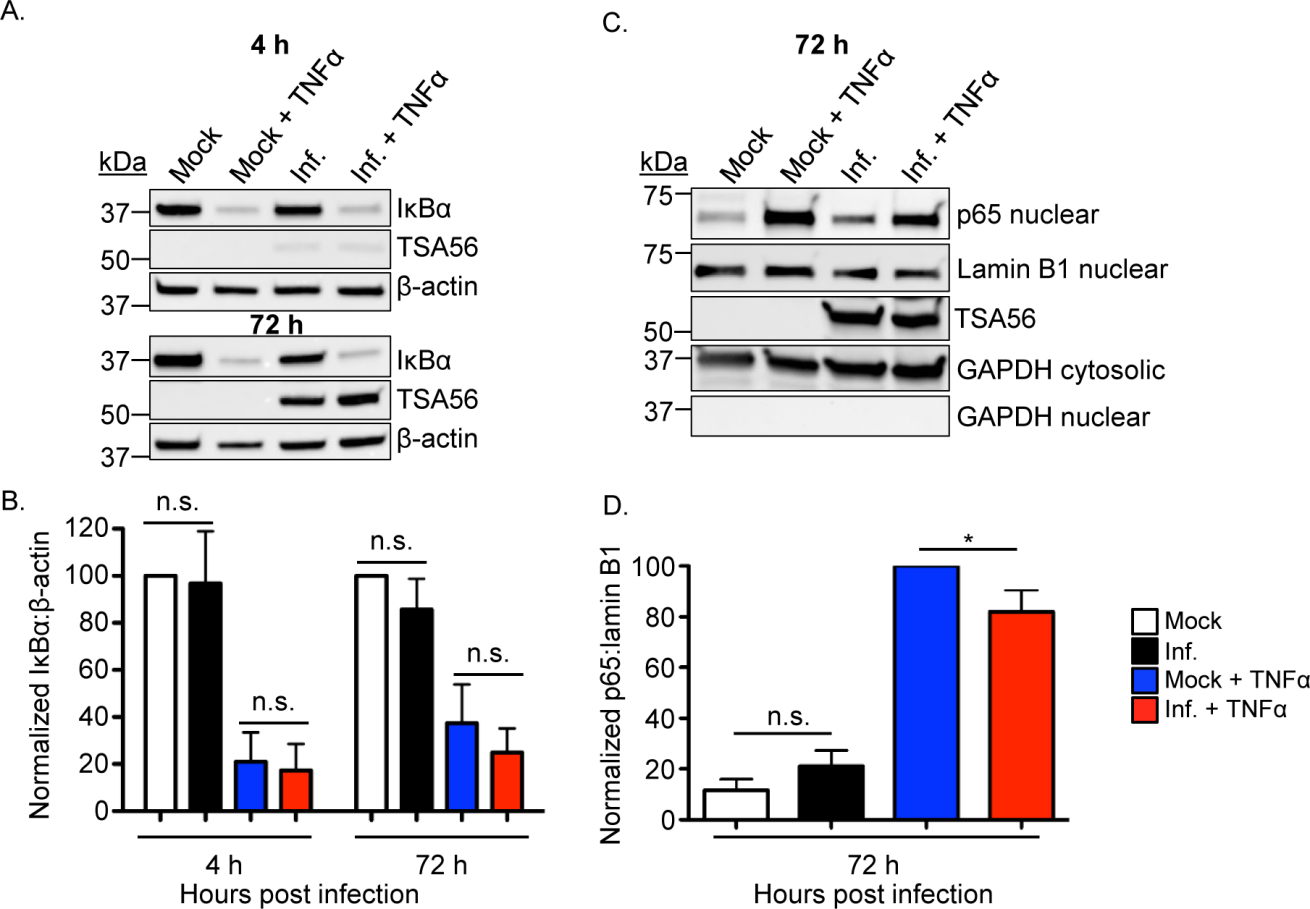
*O*. *tsutsugamushi* modulates p65 nuclear accumulation without inhibiting IκBα degradation. HeLa cells were infected with *O*. *tsutsugamushi* at an MOI of 10 (A and B) or 25 (C and D) or incubated with mock control. At 4 or 72 h, the cells were treated with TNFα or vehicle control for 30 min followed by homogenization. Whole cell lysates (A) or nuclear and cytosolic fractions (C) were analyzed by Western blotting with antibodies to IκBα, *O*. *tsutsugamushi* TSA56, and β-actin (loading control) (C) or p65, lamin B1, TSA56, and GADPH (D). Mean normalized ratios + SD of IκBα:β-actin (B) and p65:lamin B1 (D) were calculated from three separate experiments performed in (A) and (C), respectively, using densitometry. Statistically significant (*P < 0.05) values are indicated. n.s., not significant.

TNFα induced degradation of IκBα frees the p50/p65 dimer to translocate into the nucleus [27]. To assess whether the *O*. *tsutsugamushi*-mediated inhibition of p65 accumulation in the nucleus following TNFα stimulation is due to a blockage of IκBα degradation, HeLa cells were infected with *O*. *tsutsugamushi*. Mock infected cells served as controls. At 4 and 72 h, the cells were treated with TNFα or vehicle for 30 min. IκBα levels were pronouncedly reduced to a similar degree in TNFα-treated *O*. *tsutsugamushi* infected and control cells at both time points (Fig 4A and 4B). Next, the experiment was repeated except that nuclear and cytosolic fractions were analyzed following exposure to TNFα or vehicle. Screening nuclear fractions with antibodies against p65, lamin B1 (nuclear fraction loading control), and GAPDH (purity control) and cytosolic fractions with antibodies targeting TSA56 and GAPDH (cytosolic fraction loading and purity control) verified that p65 accumulation in the nuclear fraction is significantly inhibited in *O*. *tsutsugamushi* infected cells (Fig 4C and 4D).

### *O*. *tsutsugamushi* inhibits NF-κB-dependent gene transcription

**Fig 5:**
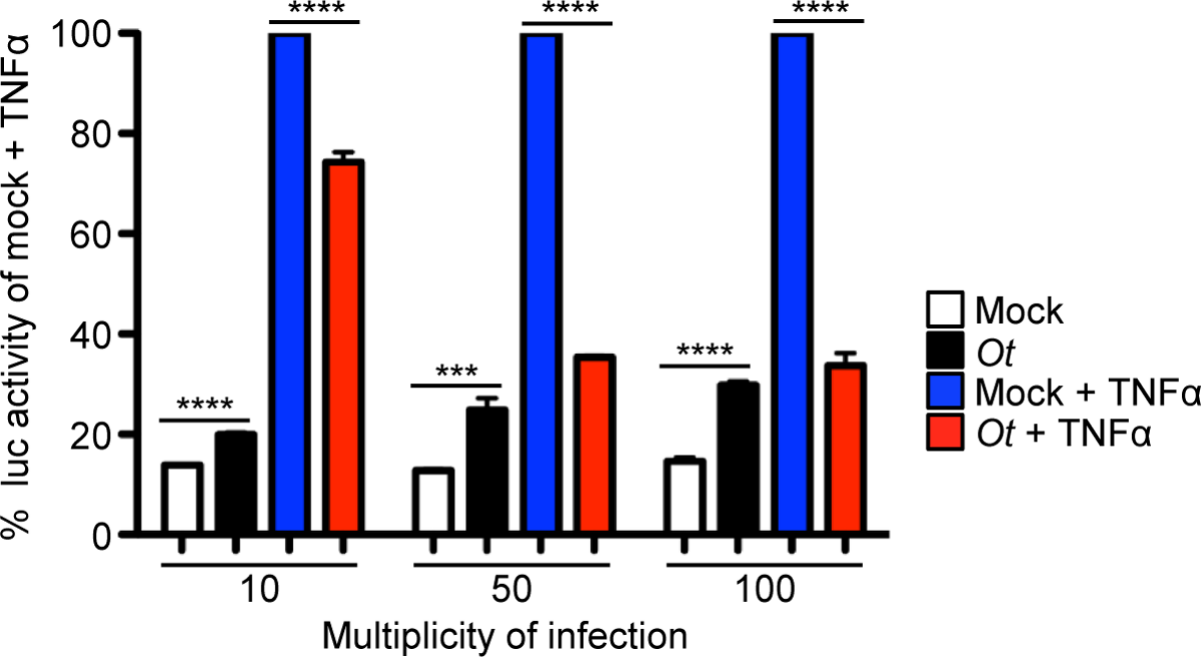
*O*. *tsutsugamushi* inhibits NF-κB-dependent gene transcription. Stably transfected HeLa cells bearing four copies of the NF-κB response element upstream of the Firefly Luciferase (Luc) gene seeded in a 96-well plate were incubated with *O*. *tsutsugamushi* at an MOI of 10, 50, or 100 or mock control for 24 h. The cells were treated with TNFα or vehicle control for 8 h to promote NF-κB-dependent gene transcription followed by the addition of lysis buffer and Luc substrate. The plate was read in a luminometer for 10 s per well to quantify Luc activity. Data are presented as the mean percentage + SD of Luc activity normalized to the Luc activity of mock control cells exposed to TNFα from triplicate samples. Statistically significant (***P < 0.001; ****P < 0.0001) values are indicated. Data are representative of three independent experiments.

To determine if *O*. *tsutsugamushi* reduces NF-κB-dependent transcriptional activation, stably transfected HeLa cells bearing four copies of the NF-κB response element upstream of the Firefly Luciferase gene were incubated with *O*. *tsutsugamushi* at MOIs of 10, 50, or 100 for 24 h. The cells were stimulated with TNFα or vehicle control followed by homogenization, addition of luciferase substrate, and measurement of the resulting luminescence signal. Compared to mock infected controls, *O*. *tsutsugamushi* stimulated a significant but low-level activation of luciferase expression (Fig 5). *O*. *tsutsugamushi* significantly reduced TNFα-stimulated luciferase production at all MOIs and in a dose-dependent manner, thereby confirming that it is capable of inhibiting NF-κB-dependent transcription.

### *O*. *tsutsugamushi* Ank1 and Ank6 inhibit p65 nuclear accumulation and translocate into the nucleus

**Fig 6:**
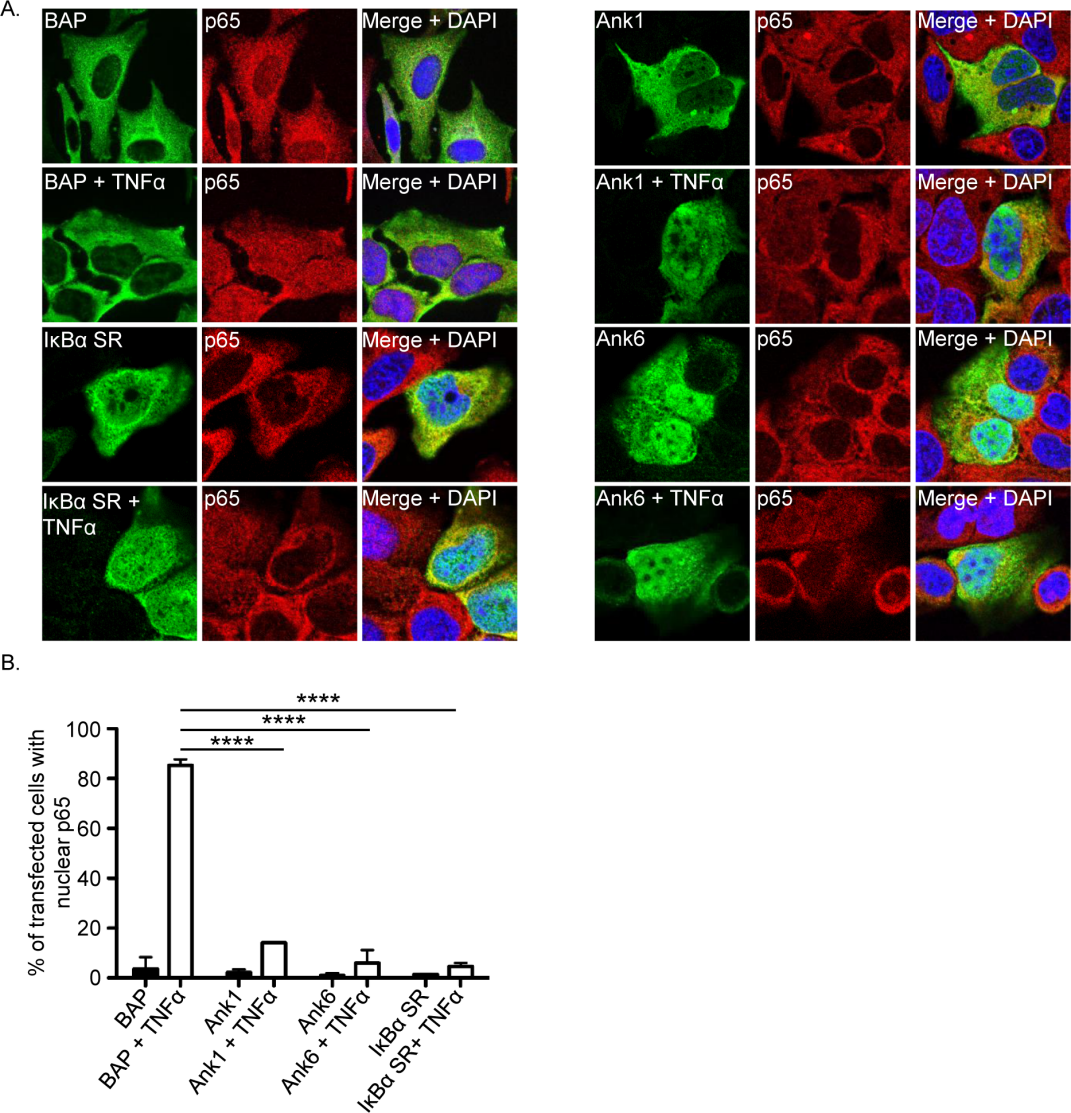
Ank1 and Ank6 translocate into the nucleus and prevent p65 nuclear accumulation. (A) HeLa cells were transfected to express Flag-tagged BAP, Ank1, Ank6, or IκBα SR. At 16 h, the cells were exposed to TNFα or vehicle control for 30 min, after which they were fixed, screened with antibodies specific for the Flag epitope and p65, and examined by confocal microscopy. (B) The mean percentage + SD of transfected cells exhibiting p65 in the nucleus was determined. Triplicate samples of 100 cells each were counted per time point. Statistically significant (****P < 0.0001) values indicated. Results are representative of three independent experiments.

*O*. *tsutsugamushi* inhibits TNFα-induced p65 accumulation in the nucleus and reduces NF-κB-dependent transcription in a manner that involves neither degradation of p65 nor inhibition of IκBα degradation and is therefore likely due to direct bacterial modulation of p65 itself. As select *O*. *tsutsugamushi* Anks have been shown to modulate diverse host cellular processes and the bacterium expresses the entire complement of *ank* genes during infection of mammalian host cells [22–25], we rationalized that one or more Anks might contribute to the pathogen’s ability to modulate p65. Knock out-complementation is not possible for *O*. *tsutsugamushi*. Moreover, even if genetic manipulation for the bacterium was feasible, it would be difficult to apply to the *anks* because many exist as multiple, near-identical copies dispersed over the chromosome [25, 40, 41]. We therefore devised a screen in which each of the 20 distinguishable *O*. *tsutsugamushi* str. Ikeda Anks N-terminally fused to either the Flag tag or GFP (green fluorescence protein) [25] were examined by confocal microscopy for the ability to inhibit TNFα-induced p65 accumulation in the nucleus when ectopically expressed in HeLa cells. Flag-BAP (bacterial alkaline phosphatase) and GFP alone served as negative controls. As a positive control for p65 inhibition, cells were transfected to express a Flag-tagged version of the IκBα super repressor (IκBα SR), which has Ala substitutions for Ser32 and Ser36 to prevent its phosphorylation and degradation [42]. In the absence of TNFα, p65 was devoid from the nuclei of nearly all transfected cells regardless of the construct (Figs 6 and S3), verifying that neither the transfection procedure nor any of the ectopically expressed proteins stimulated the NF-κB response. Following exposure to TNFα, p65 was detected in the nuclei of more than 80% of cells expressing Flag-BAP or GFP and almost none of the cells expressing Flag-IκBα SR. Of the 20 Anks examined, only Ank1 (Ank1_02; OTT_0753)[25, 41] and Ank6 (Ank6_02; OTT_1149)[25, 41] inhibited TNFα-stimulated p65 nuclear accumulation to phenocopy events associated with *O*. *tsutsugamushi* infection (Figs 6, S3 and S4).

**Fig 7:**
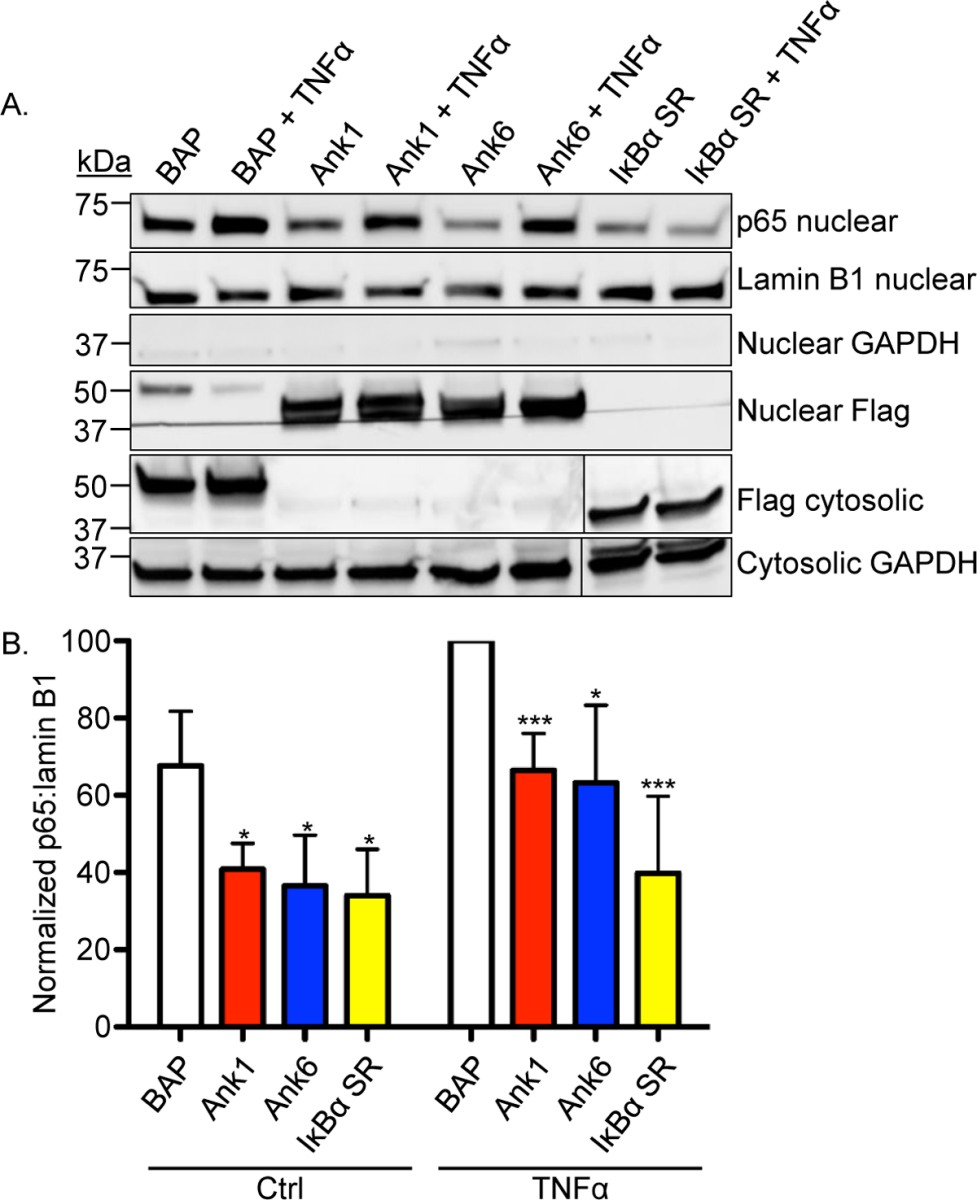
Ank1 and Ank6 translocate into the nucleus. (A) HeLa cells were transfected to express Flag-tagged BAP, Ank1, Ank6, or IκBα SR. At 16 h, they were treated with TNFα or vehicle control (Ctrl) for 30 min. Western blotted nuclear or cytosolic fractions were screened with antibodies against p65, lamin B1, GAPDH, or the Flag epitope. (B) Mean normalized ratios + SD of p65:lamin B1 densitomery signals were calculated from three separate experiments performed in (A). Statistically significant values (*P < 0.05; ***P < 0.001) are indicated. Data presented are representative of three separate experiments.

To complement the immunofluorescence screen, nuclear and cytosolic fractions of HeLa cells transfected to express Flag-tagged Ank1, Ank6, IκBα SR, or BAP were subjected to Western blot and densitometry analyses. Consistent with the immunofluorescence results (Fig 6), the amount of p65 present in the nuclear fractions was significantly reduced in cells expressing Flag-tagged Ank1, Ank6, and IκBα SR as compared to Flag-BAP, whether exposed to TNFα or not (Fig 7A and 7B). Notably, Flag-tagged Ank1 and Ank6 were present in the nuclear fractions (Fig 7A). Flag-Ank1 and Flag-Ank6 translocated into the nucleus comparably well in the presence and absence of TNFα (Fig 7A and 7B). Taken together, these data demonstrate that Ank1 and Ank6 are each capable of translocating into the nucleus and inhibiting TNFα-stimulated p65 nuclear accumulation.

### Ank1 and Ank6 sequence attributes

Ank1 exists as two paralogs in the *O*. *tsutsugamushi* str. Ikeda genome, Ank1_01 and Ank1_02, the former of which is truncated, lacks an ankyrin repeat domain, and is homologous to the non-ankyrin repeat region of Ank1_02. Ank6 exists as four paralogs that exhibit 99.1 to 100% amino acid identity with each other. The respective Ank1 and Ank6 paralogs are not tandemly arranged, but are instead distributed over the chromosome [25, 41]. Ank1_02 and Ank6_02, the representative Ank1 and Ank6 paralogs chosen for this study, are hereafter simply referred to as Ank1 and Ank6, respectively. Ank1 and Ank6 are annotated to contain three and four ankyrin repeats, respectively, and C-terminal F-box and PRANC (Pox proteins Repeat of ANkyrin, C-terminal; consists of an F-box and flanking upstream amino acids [43]) domains (S5 Fig and S1 Table). Ank1 residues 122 to 157, which were not originally annotated to constitute an ankyrin repeat [41], exhibit 41.7% identity to Ank6 residues 122 to 158 that are predicted to form an ankyrin repeat. Therefore, Ank1 amino acids 122 to 157 might comprise an ankyrin repeat that had not been properly annotated. Ank1 and Ank6 bear 58.6% identity over the entirety of their sequences, 61.9% identity in their N-termini, 37.2% identity in their ankyrin repeat domains, 76.9% identity in their PRANC domains, 76.2% identity in their F-boxes, and 55.6% identity in their C-termini (S1 Table). The region of greatest amino acid identity between the two proteins (84.1%) occurs at the intervening sequence region (ISR) between the ankyrin repeat region and PRANC domain, which corresponds to Ank1 residues 167 to 201 and Ank6 residues 168 to 202. *In silico* analyses did not detect a canonical NLS in either protein. Eukaryotic ankyrin repeat domains can facilitate nuclear localization through RanGDP binding at two consecutive ankyrin repeats provided that a hydrophobic residue is located at the thirteenth position in each of the two repeats [44]. Evidence suggests that this phenomenon might be recapitulated by the nucleotropic effector, AnkA, of *Anaplasma phagocytophilum* [45], which is in the Order *Rickettsiales* with *O*. *tsutsugamushi*. However, neither Ank1 nor Ank6 meet this criterion (S5 Fig). Nuclear export sequences (NES) tend to be leucine-rich [46–48]. According to the NetNES 1.1 server (http://www.cbs.dtu.dk/services/NetNES/) [48], Ank6 residues 143 to 161 comprise a potential leucine-rich nuclear export sequence (NES). The corresponding amino acids (142 to 160) of Ank1 are not predicted to form an NES (S5 Fig and S1 Table).

### Ank1 and Ank6 interact with endogenous p65 but not IκBα

**Fig 8:**
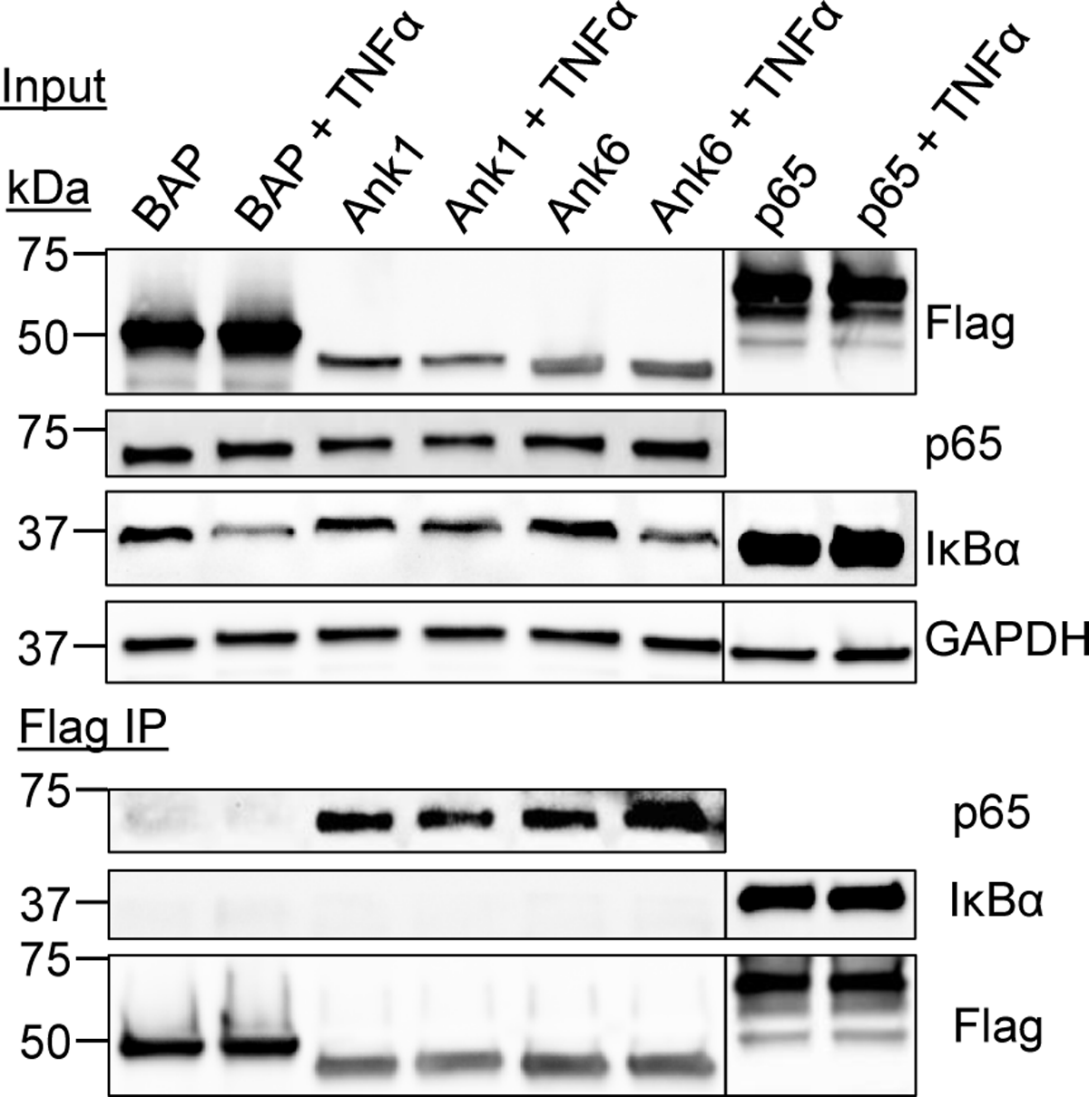
Ank1 and Ank6 each interact with p65. HeLa cells ectopically expressing Flag-tagged BAP, Ank1, or Ank6 and were treated with TNFα or vehicle for 30 min. Whole cell lysates were incubated with Flag antibody-conjugated agarose beads to immunoprecipitate (IP) Flag-tagged proteins and their interacting proteins. Resulting Western blots were probed with p65 antibody to determine if any Flag-tagged protein co-immunoprecipitated endogenous p65. As a control to ensure that the conditions allowed for p65 and IκBα to interact, a parallel experiment was performed in which lysates of TNFα or vehicle control stimulated Flag-p65 expressing HeLa cells were incubated with the Flag antibody coated beads followed by Western blot analysis of the resulting eluate with antibody against endogenous IκBα. Expression of each Flag-tagged protein of interest was confirmed by subjecting input lysate to Western blotting using Flag antibody. Data presented are representative of three independent experiments.

To determine if Ank1 or Ank6 is capable of interacting with p65, HeLa cells expressing Flag-tagged Ank1, Ank6, or BAP were exposed to TNFα or vehicle, lysed, and incubated with Flag affinity agarose beads. Immunoprecipitated Flag-tagged proteins and any co-immunoprecipitated interacting partners were Western-blotted. Probing Western blots of input lysates confirmed that all Flag-tagged bait proteins were expressed (Fig 8). Screening Flag pulldown samples with p65 antibody revealed that Flag-tagged Ank1 and Ank6, but not BAP co-immunoprecipitated endogenous p65. Flag-Ank1 and Flag-Ank6 interacted with p65 whether TNFα was present or not, but failed to precipitate endogenous IκBα in either condition. Flag-p65 co-immunoprecipitated IκBα, which verified that conditions allowed for p65 and IκBα to interact. Therefore, Ank1 and Ank6 are capable of interacting with p65, but neither directly nor indirectly interacts with IκBα.

### Ank1 and Ank6 co-immunoprecipitate endogenous importin β1 and exportin 1

**Fig 9:**
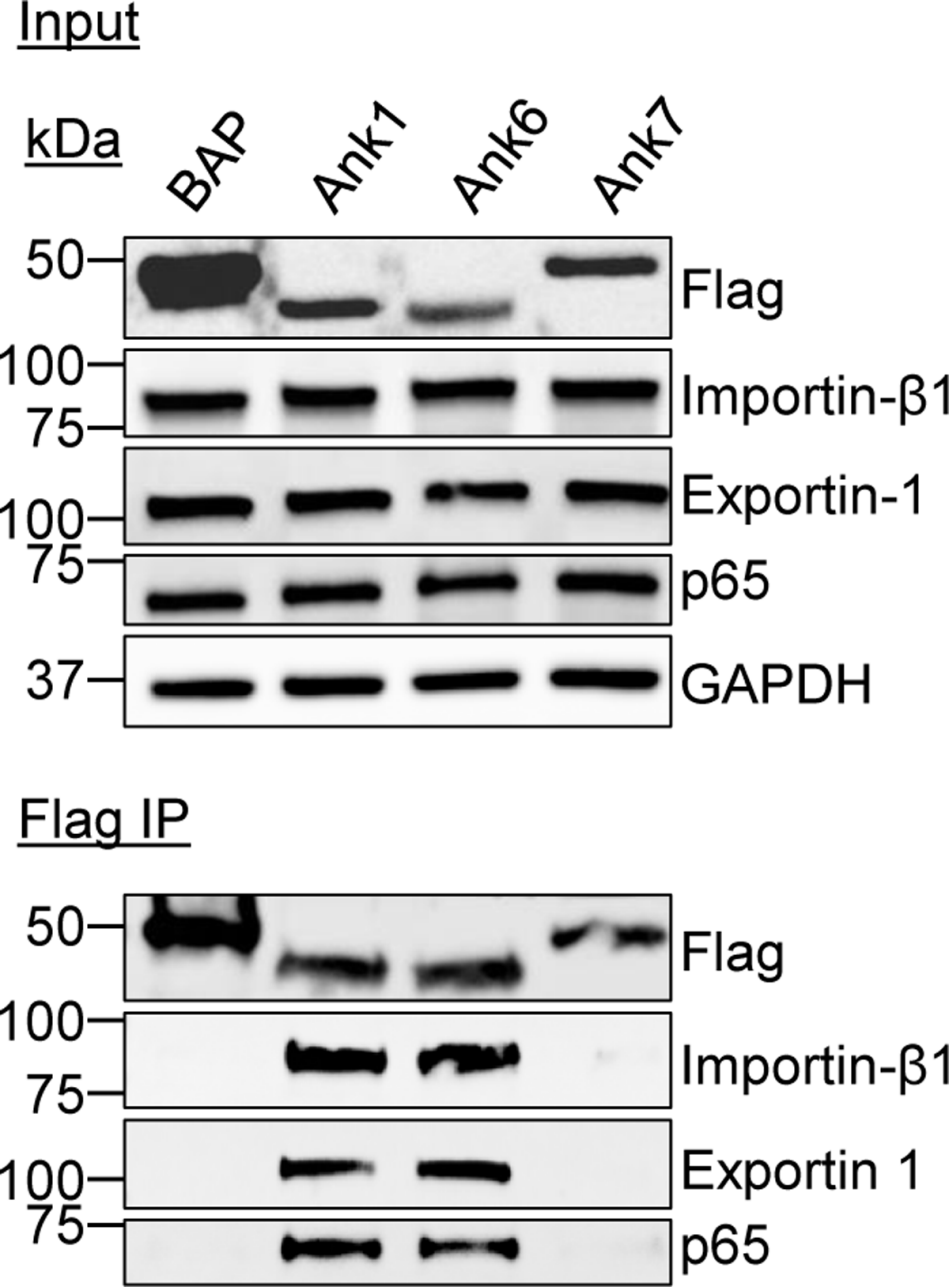
Ank1 and Ank6 each interact with importin β1 and exportin 1. Whole cell lysates of HeLa cells expressing Flag-tagged BAP, Ank1, Ank6, or Ank7 were incubated with Flag antibody-conjugated agarose beads to immunoprecipitate Flag-tagged proteins and their interacting proteins. Resulting Western blots were probed with antibodies specific for importin β1, exportin 1, and p65. Expression of each Flag-tagged protein of interest was confirmed by subjecting input lysate to Western blotting using Flag antibody. Data presented are representative of three independent experiments.

The mechanisms by which Ank1 and Ank6 translocate into the nucleus and prevent p65 nuclear accumulation are unknown. Importin β1 and exportin 1 are two proteins of the karyopherin-β family that regulate import and export of nuclear cargo, respectively, including NF-κB [46, 49]. In the classic nuclear import pathway, cargo bearing an NLS is bound by an importin α protein, which, in turn, is bound by an importin β protein that is essential for translocation of the ternary complex through the nuclear pore [50]. Importin β1 is the most well characterized karyopherin-β protein and has a conserved function in NLS-mediated cargo import into the nuclei of animals, plants, and eukaryotic microbes [51]. Because importin β1 is capable of dimerizing with most of the six different importin α proteins [46], the most direct means for assessing if Ank1 or Ank6 potentially exploits the classic nuclear import pathway was to determine if they are capable of interacting with importin β1. Exportin 1, also known as CRM1 (chromosome region maintenance 1), is a ubiquitous receptor protein that binds to nuclear export signals on a variety of cargoes to direct their translocation from the nucleus to the cytosol [46]. We therefore also examined if Ank1 and Ank6 are capable of binding exportin 1. HeLa cells transfected to express Flag-tagged Ank1, Ank6, or BAP were subjected to immunoprecipitation using Flag affinity agarose resin. Western blots of eluted immunoprecipitated complexes were probed with antibodies specific for endogenous importin β1, exportin 1, and p65 as a positive control. All three proteins were co-immunoprecipitated by Flag-Ank1 and Flag-Ank6, but not Flag-BAP even though Flag-BAP was expressed and recovered at a considerably higher abundance (Fig 9). Thus, Ank1 and Ank6 are each capable of interacting with importin β1 and exportin 1. To verify that the abilities of Ank1 and Ank6 to bind p65, importin β1, and exportin 1 were not simply due to their possessing an ankyrin repeat domain, Flag-tagged *O*. *tsutsugamushi* Ank7_02 (OTT_1509) was included as a negative control because the effector does not inhibit p65 nuclear accumulation (S3 Fig) and remains in the host cell cytosol when ectopically expressed [25]. Flag-Ank7_02 failed to co-immunoprecipitate importin β1, exportin 1, and p65 (Fig 9).

### Ank1 and Ank6 translocation into the nucleus is dependent on importin β1 activity

**Fig 10:**
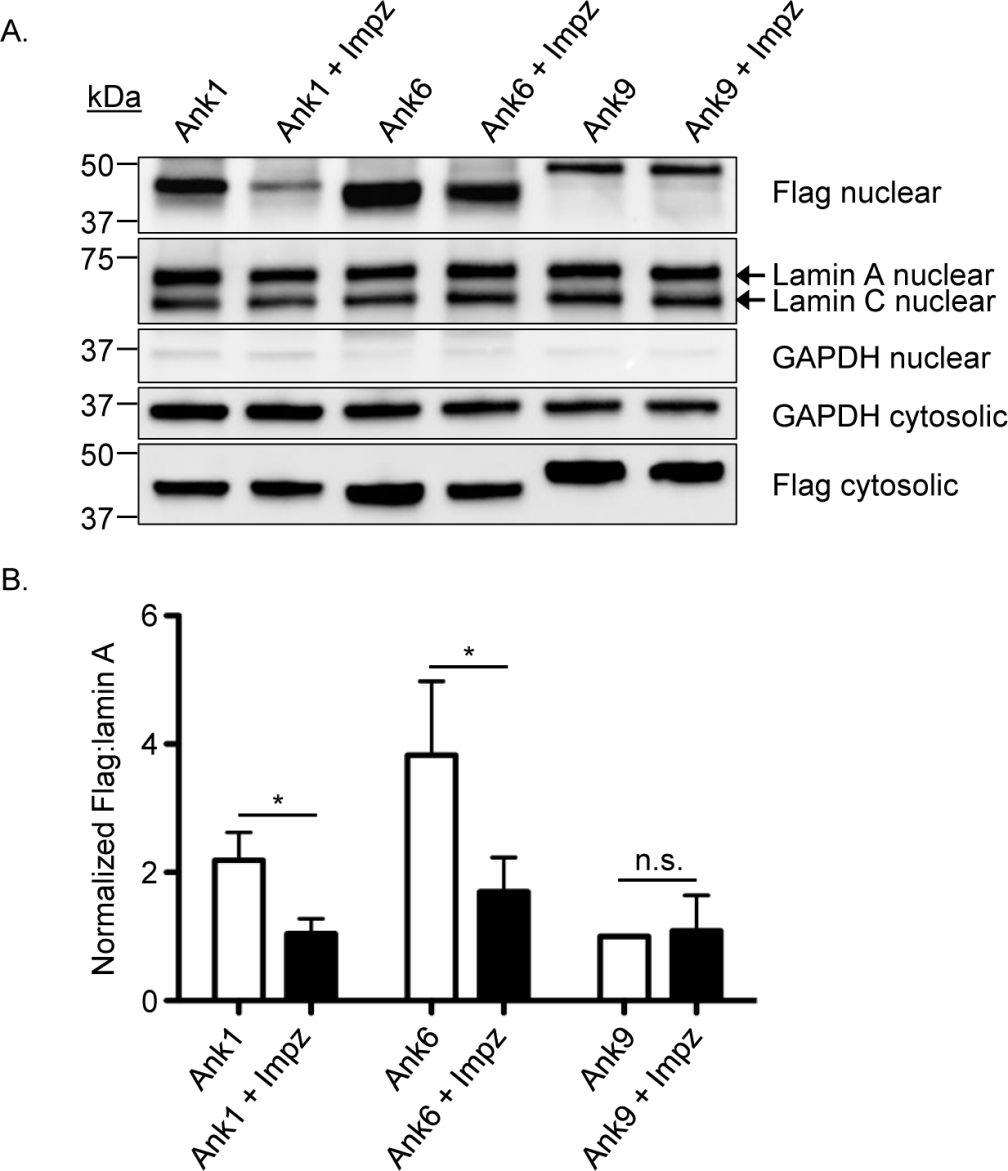
Ank1 and Ank6 require importin β1 activity to optimally translocate into the nucleus. (A) HeLa cells were transfected to express Flag-tagged Ank1, Ank6, or Ank9. At 16 h, the cells were treated with importazole or vehicle for 3 h. Following lysis and nuclear fractionation, Western blotted nuclear or cytosolic fractions were screened with antibodies against the Flag epitope, lamin A/C, and GAPDH. (B) Mean ratios + SD of Flag:lamin A densitometric signals from three separate experiments were normalized to Flag-Ank9 to account for the low level background nuclear accumulation of the ectopically expressed Anks. Statistically significant (*P < 0.05) values are indicated. n.s., not significant. Data presented are representative of three independent experiments.

Given that importin β1 is essential for the import of cargo into the nucleus from the cytosol [50], we directly assessed if this protein’s activity is critical for Ank1 and Ank6 nuclear transport. HeLa cells expressing Flag-tagged Ank1, Ank6, or Ank9 were treated with importazole, a small molecule that selectively inhibits importin β1 to block nuclear import of importin β1-dependent proteins [52], for 3 h. Ank9 was utilized as a negative control because it targets Golgi-to-ER retrograde traffic and, when ectopically expressed N-terminally fused to the Flag tag, does not robustly accumulate in the nucleus [24]. Western blot and densitometry analyses were performed to assess for Flag-Ank protein levels in the nucleus and cytosol. Lamin A/C and GAPDH antibodies were used as a nuclear loading control and to confirm fraction purity, respectively. Flag-Ank1 and Flag-Ank6 were detected at much higher levels in the nucleus than Flag-Ank9 (Fig 10). Importazole treatment reduced Flag-Ank1 and Flag-Ank6 nuclear levels by 52.3% and 55.6%, respectively, but failed to reduce the low background nuclear level of Flag-Ank9. Even though importazole reduced cytosolic levels of all three Flag-tagged proteins (Figs 10A and S6), the reduction in Flag-Ank1 and Flag-Ank6 cytosolic levels was not as pronounced as for their reduction in the nucleus. These data demonstrate that Ank1 and Ank6, but not Ank9 depend on an importazole-sensitive pathway to optimally traffic into the nucleus and the slightly reduced level of Flag-tagged Ank1 and Ank6 in importazole treated cells does not account for the marked decrease in their nuclear levels. Overall, it can be concluded that both Ank1 and Ank6 exploit importin β1 activity for optimal translocation into the nucleus.

### Ank1 and Ank6 rely on exportin 1 function to promote p65 export from the nucleus

**Fig 11:**
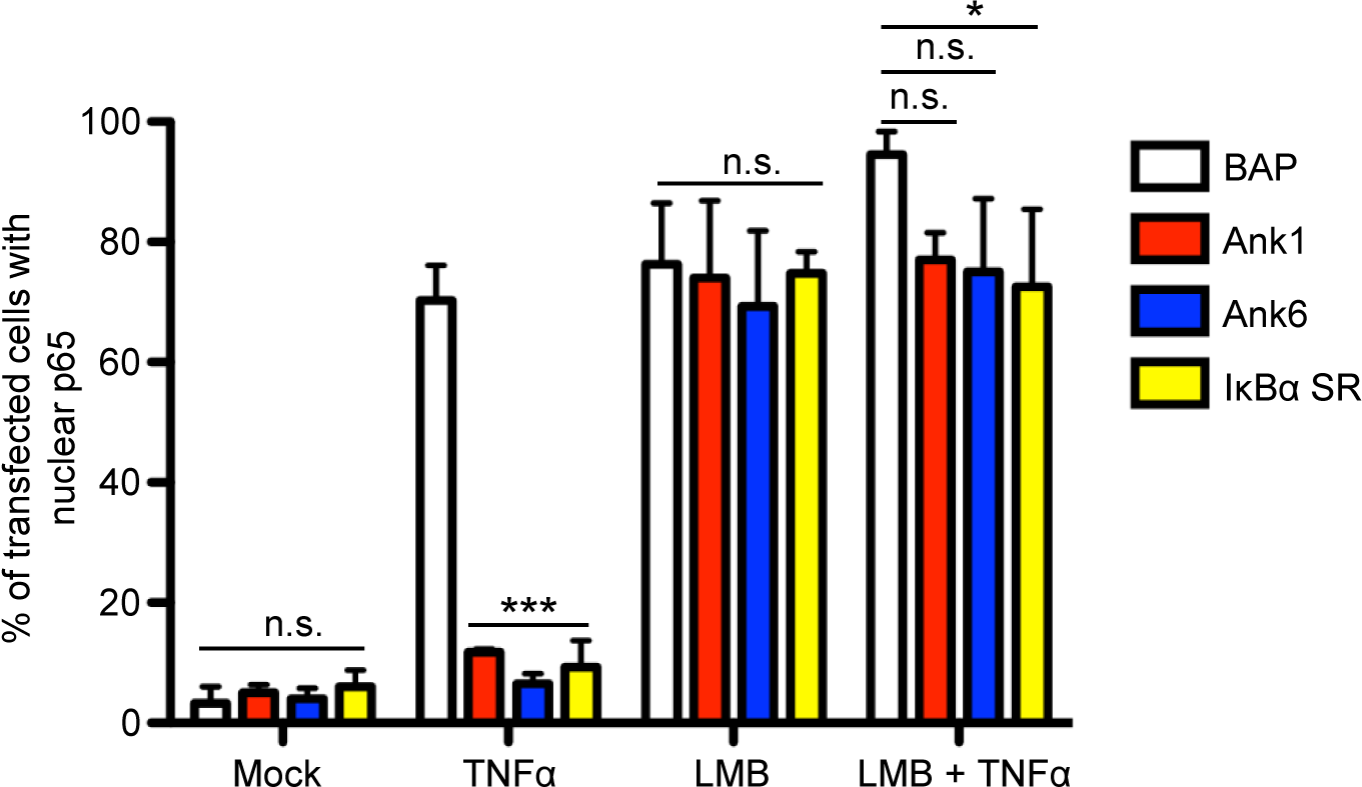
Ank1 and Ank6 promote p65 removal from the nucleus in an exportin 1-dependent manner. HeLa cells were transfected to express Flag-tagged BAP, Ank1, Ank6, or IκBα SR. At 16 h, the cells were treated with LMB or vehicle control for 1 h. The media was replaced with media containing TNFα or vehicle for 30 min. The cells were then fixed, screened with antibodies specific for the Flag epitope and p65, and examined by confocal microscopy. Representative fluorescence images are presented in S7 Fig. The mean percentage + SD of cells exhibiting p65 in the nucleus was determined. Quadruplicate samples of 100 cells each were counted per time point. Statistically significant (*P < 0.05; ***P < 0.001) values are indicated. n.s., not significant. Results are representative of three independent experiments.

The abilities of Ank1 and Ank6 to prevent nuclear accumulation of p65 could either be due to inhibition of p65 import into or promotion of p65 export from the nucleus. To resolve this discrepancy, it was examined if Ank1 and Ank6 depend on exportin 1 activity to inhibit p65 nuclear accumulation. Exportin 1 directs NF-κB export from the nucleus by binding to an NES on IκBα bound to NF-κB and translocates the IκBα:NF-κB complex to the cytosol [46]. Leptomycin B (LMB) is a fungal metabolite that covalently binds to exportin 1, irreversibly blocking its ability to bind the NES of IκBα (and other nuclear proteins), to prevent export of the IκBα:NF-κB complex [49, 53]. HeLa cells expressing Flag-tagged Ank1, Ank6, IκBα SR, or BAP were treated with LMB or control followed by the addition of TNFα or vehicle. The cells were examined for the presence of p65 in the nucleus using confocal microscopy. Whereas Flag-tagged Ank1, Ank6, and IκBα SR abrogated TNFα-stimulated p65 nuclear accumulation, LMB eliminated this phenomenon (Figs 11 and S7). Thus, Ank1 and Ank6 co-opt exportin 1 activity to promote p65 export from the nucleus.

### Ank1 and Ank6 inhibit NF-κB-dependent transcriptional activation in an exportin 1-independent manner

**Fig 12:**
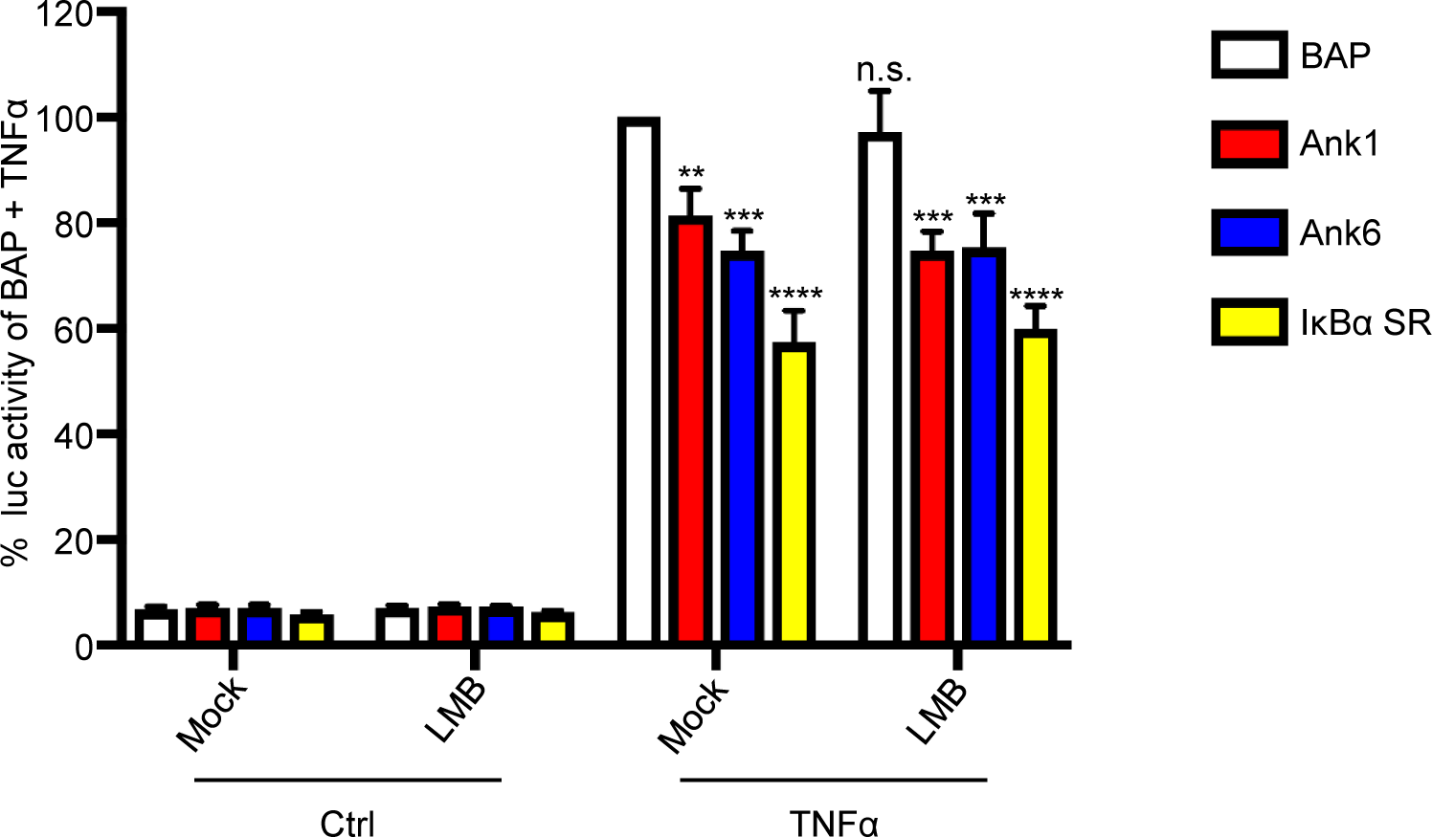
Ank1 and Ank6 inhibit NF-κB-dependent transcriptional activation in an exportin 1-independent manner. Stably transfected HeLa cells bearing four copies of the NF-κB response element upstream of the Luc gene were transiently transfected to express the indicated Flag-tagged proteins for 16 h. The cells were treated with LMB or vehicle control in the presence or absence of TNFα for 4 h followed by lysis and mixing with Luc substrate. Data are presented as the mean percentage + SD of Luc activity normalized to the Luc activity of control cells expressing Flag-BAP and exposed to TNFα from triplicate samples. Statistically significant (**P < 0.01; ***P < 0.001; ****P<0.0001) values are indicated. n.s., not significant. Results are representative of three independent experiments.

Because *O*. *tsutsugamushi* inhibits NF-κB-dependent gene transcription, it was examined if Ank1 and Ank6 copy this phenomenon and do so in an LMB-sensitive manner. HeLa cells having four copies of the NF-κB response element upstream of the Firefly Luciferase gene were transiently transfected to express Flag-tagged Ank1, Ank6, IκBα SR, or BAP. The cells were incubated with LMB or control in the presence or absence of TNFα. The cells were lysed, luciferase substrate was added, and the luminescence signal was recorded. Background luminescence levels in the absence of TNFα were similar among all samples, indicating that the transfection procedure, protein overexpression, and LMB did not activate transcription from the NF-κB response element (Fig 12). Relative to Flag-BAP, cells expressing Flag-tagged Ank1, Ank6, or IκBα SR each exhibited reduced luciferase activity whether LMB was present or not. Thus, like the IκBα SR, Ank1 and Ank6 antagonize the NF-κB transcriptional response, but do not require exportin 1 activity to do so.

### *O*. *tsutsugamushi* promotes p65 nuclear export in an exportin 1-dependent manner, but inhibits NF-κB-dependent transcriptional activation in an exportin 1-independent manner

**Fig 13:**
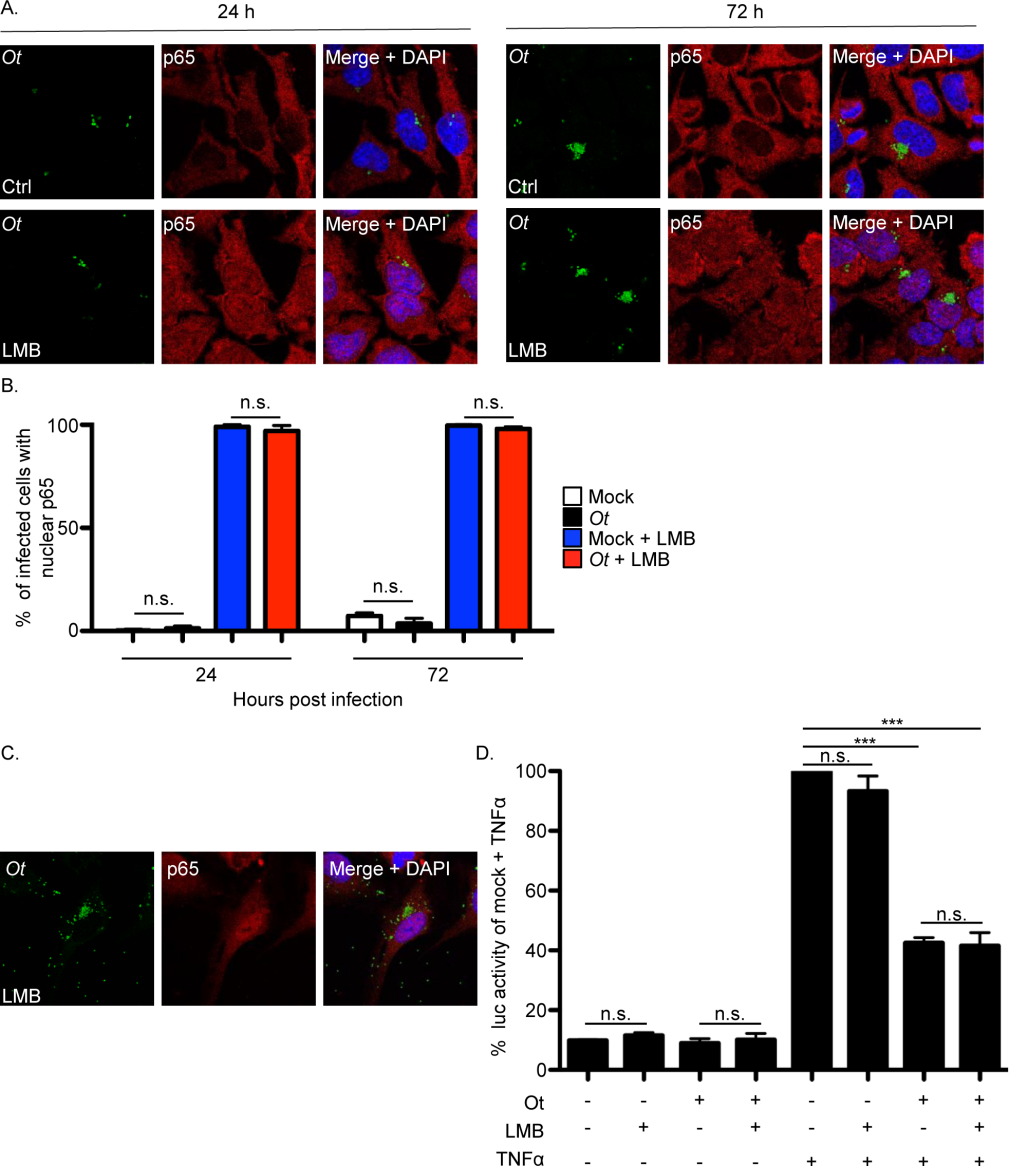
Exportin 1 activity is critical for *O*. *tsutsugamushi* to promote p65 export from the nucleus, but not for the bacterium to inhibit NF-κB-dependent transcriptional activity. (A and B) *O*. *tsutsugamushi* mediates p65 nuclear export in an LMB-sensitive manner. HeLa cells were infected with *O*. *tsutsugamushi* at an MOI of 10 for 24 h or 72 h. Next, the cells were treated with LMB or vehicle control for 1 h after which they were fixed, screened with antibodies against *O*. *tsutsugamushi* TSA56 (*Ot*) and p65, and examined by confocal microscopy. (A) Representative fluorescence images of cells viewed for *Ot*, p65, and merged images plus DAPI for infected cells treated with LMB or vehicle control (Ctrl) are presented. (B) The mean percentage + SD of cells exhibiting p65 in the nucleus was determined for each condition. Triplicate samples of 100 cells each were counted per time point. n.s., not significant. Results are representative of three separate experiments. (C and D) *O*. *tsutsugamushi* inhibits NF-κB-dependent transcriptional activation in an LMB-insensitive manner. Stably transfected HeLa cells bearing four copies of the NF-κB response element upstream of the Luc gene were infected with *O*. *tsutsugamushi* for 24 h at an MOI of 10 after which they were incubated with LMB or vehicle control in the presence or absence of TNFα for 8 h. (C) Representative fluorescence images of LMB-treated infected cells viewed for TSA56 (*Ot*), p65, and merged images plus DAPI. (D) The cells were lysed followed by mixing with Luc substrate. Data are presented as the mean percentage + SD of Luc activity normalized to the Luc activity of uninfected vehicle control treated cells that were exposed to TNFα from triplicate samples. Statistically significant (***P < 0.001) values are indicated. n.s., not significant. Results are representative of three independent experiments.

To determine if *O*. *tsutsugamushi* mediated reduction of p65 in the nucleus is LMB-sensitive, HeLa cells that had been infected for 24 or 72 h were treated with the inhibitor and examined. In contrast to vehicle control treated infected cells, abundant immunolabeled p65 was detected in the nuclei of LMB treated infected cells (Fig 13A and 13B). Next, the NF-κB-dependent luciferase reporter HeLa cells were infected with *O*. *tsutsugamushi* for 24 h after which they were incubated with LMB or control in the presence or absence of TNFα. LMB blocked the ability of the bacterium to promote reduction of p65 in the nucleus (Fig 13C), but not its ability to inhibit NF-κB-dependent luciferase expression (Fig 13D). These results indicate that *O*. *tsutsugamushi* prevents nuclear accumulation of p65 by promoting its export from the nucleus in an exportin 1-reliant manner, but does not require p65 nuclear export to inhibit NF-κB-dependent transcription. In other words, *O*. *tsutsugamushi*, like Ank1 and Ank6, antagonizes NF-κB-dependent transcriptional activation even when NF-κB remains in the nucleus.

### The ankyrin repeat domains and ISR of Ank1 and Ank6 are essential for optimal interaction with p65, exportin 1, and importin β1

**Fig 14:**
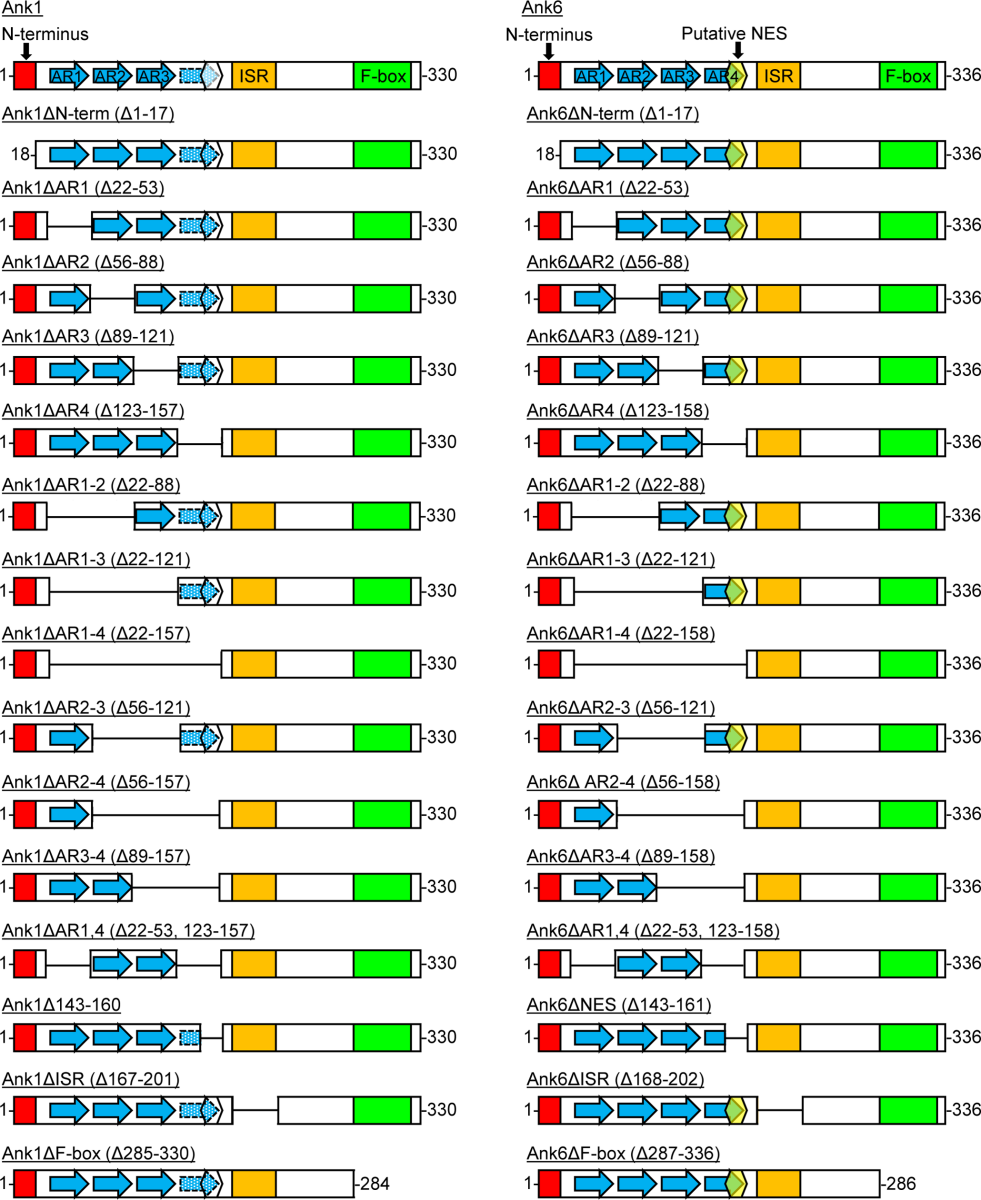
Schematics of Ank1 and Ank6 mutant proteins. Schematics of full-length wild-type Ank1 and Ank6 are presented atop the left and right column, respectively, below each of which are schematics in which deleted (△) amino acids and corresponding domains are indicated. The amino termini consisting of residues 1 through 17 are represented by red boxes. Each annotated ankyrin repeat (AR) is denoted by a blue-filled arrow bordered by a solid line. The arrow filled with white dots over a blue background and bordered by a hatched line corresponds to the potential AR that is not annotated as an AR but exhibits 41.7% identity with Ank6 AR4. The yellow hexagon in Ank6 corresponds to a putative nuclear export sequence (NES) that is found in the C-terminal region of AR4. The corresponding amino acids of Ank1 (143–160) that align with the Ank6 putative NES but are not predicted to be an NES are denoted by a white hexagon. An orange box demarcates the intervening sequence region (ISR) corresponding to Ank1 residues 167 to 201 and Ank6 168 to 202. The C-terminal F-box of each Ank is indicated by a green box.

**Fig 15:**
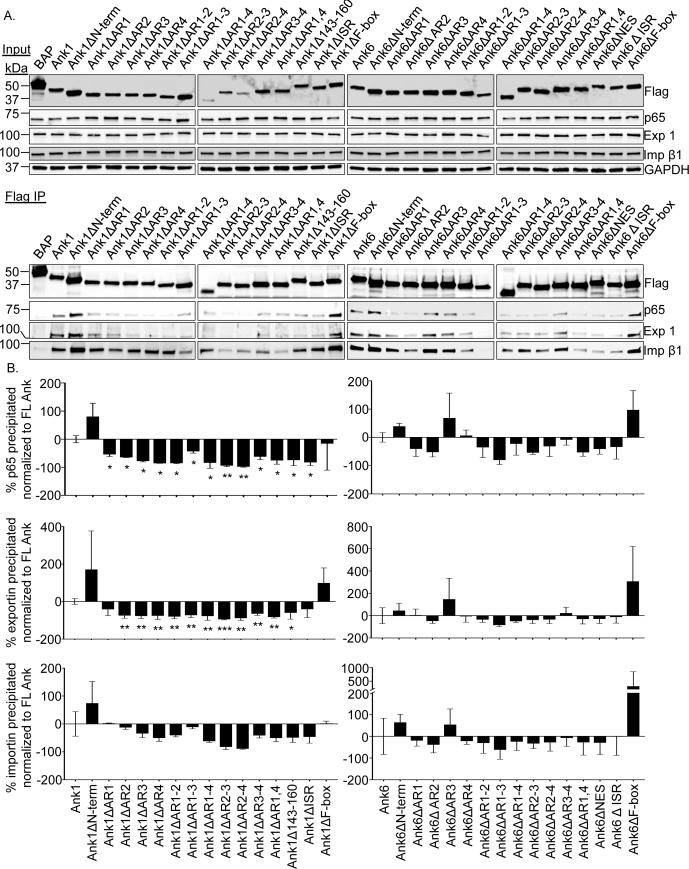
Identification of the Ank1 and Ank6 domains that are essential for the ability to co-immunoprecipitate p65, importin β1, and exportin 1. Whole cell lysates of HeLa cells expressing the indicated Flag-tagged proteins were incubated with Flag antibody-conjugated agarose beads to immunoprecipitate Flag-tagged proteins and their interacting proteins. (A) Western blots of input lysates and immunoprecipitates were probed with p65, exportin 1 (Exp 1), and importin β1 (Imp β1) antibodies. Expression of each Flag-tagged protein of interest was confirmed by subjecting input lysate to Western blotting using Flag antibody. GAPDH immunoblotting of input lysates served as a loading control. (B) Mean + SD of co-immunoprecipitated p65, exportin 1, or importin β1. Values are presented as the percent change in densitometric signal relative that for each respective protein of interest that was coimmunoprecipitated by Flag-tagged Ank1 or Ank6. Data presented were calculated from three separate experiments formed in (A). Statistically significant (*P < 0.05; **P < 0.01) values are indicated.

The Ank1 and Ank6 domains that are critical for their abilities to interact with p65, importin β1, and exportin 1; to traffic into the host cell nucleus; and to inhibit p65 accumulation in the nucleus are undefined. As a first step in identifying such domains, 30 constructs were generated for expressing N-terminally Flag-tagged versions of either protein lacking the N-terminus, one or every possible combination of multiple ankyrin repeats (including the potential Ank1 ankyrin repeat that aligns with the fourth ankyrin repeat of Ank6; S5 Fig and S1 Table; hereafter referred to as Ank1 ankyrin repeat four), putative NES of Ank6 (or corresponding Ank1 amino acids 143 to 160), ISR, or F-box (Fig 14). This approach has proven effective for identifying protein-protein interaction domains of many other microbial F-box containing Ank proteins [22, 45, 54–64]. Next, the abilities of these proteins to co-immunoprecipitate p65, importin β1, and exportin 1 were examined. HeLa cells transfected to express Flag-tagged Ank1, Ank6, truncation mutants thereof, or BAP were subjected to Flag affinity agarose resin immunoprecipitation. Probing Western blots of input lysates with Flag epitope, p65, importin β1, and exportin 1 antibodies confirmed that all four proteins of interest were present in every sample (Fig 15). GAPDH served as a loading control. Western blot and densitometry analyses of eluted immunoprecipitated complexes confirmed previous results (Figs 8 and 9) that Flag-tagged Ank1 and Ank6, but not BAP precipitate p65, importin β1, and exportin 1 (Fig 15). Flag-Ank1 and Flag-Ank6 proteins lacking the N-terminus or F-box pulled down all three targets comparable to or better than their full-length counterparts, demonstrating that these regions are irrelevant for mediating such interactions.

Flag-Ank1 proteins lacking any single ankyrin repeat, multiple combinations thereof, or the ISR were compromised in the ability to interact with p65 (Fig 15). Most defective were Flag-Ank1△AR (ankyrin repeat)2-3 and Flag-Ank1△AR2-4. These results suggest that, while an intact ankyrin repeat domain is required for Ank1 to bind p65, ankyrin repeats two and three functioning in tandem are the most critical. Flag-Ank1△143–160 also exhibited reduced interaction with p65, likely due to the fact that ankyrin repeat 4 was disrupted. Flag-Ank6 proteins that lacked ankyrin repeat 1 or 2, any combination of two or more ankyrin repeats, the putative NES within ankyrin repeat four, or the ISR exhibited varied reduction in the ability to co-immunoprecipitate p65.

When the Flag-Ank1 and Flag-Ank6 proteins were examined for the proficiency to pull down exportin 1, the results were similar to those obtained for p65 (Fig 15). It is unlikely that the same domains of Ank1 and Ank6 form interactions with p65 and exportin 1, as they would otherwise compete with each other. Given that exportin 1 is a natural binding partner of p65 [46], the observed overlap in interaction profiles could be due to Flag-tagged Ank1 and Ank6 indirectly co-immunoprecipitating exportin 1 through direct interactions with p65. Overall, these results demonstrate that an intact ankyrin repeat domain is key for Ank1 or Ank6 to optimally bind p65 and exportin 1, multiple ankyrin repeats are cooperatively involved, and the ISR of both proteins also contributes to these interactions.

With the exception of ankyrin repeat 1, Flag-tagged Ank1 required an intact ankyrin repeat domain and the ISR to optimally interact with importin β1 (Fig 15). Flag-Ank6 proteins’ optimal interactions with importin β1 also involved the ankyrin repeat region, except for ankyrin repeat 3. Notably, ankyrin repeat 3 alone was dispensable for Flag-Ank6 to interact with p65, exportin 1, and importin β1. Overall, it can be concluded that, while the ankyrin repeat-ISR regions of Ank1 and Ank6 are necessary for optimally interacting with p65, exportin 1, and importin β1, the responsible sub-domains thereof are distinct between the two effectors. Also, because certain truncations retained the ability to interact with the targets it can be concluded that loss of interaction is specific and not merely due to deleting any portion of Ank1 or Ank6.

### Ank1 and Ank6 domains that are critical for optimal translocation into the nucleus

**Fig 16:**
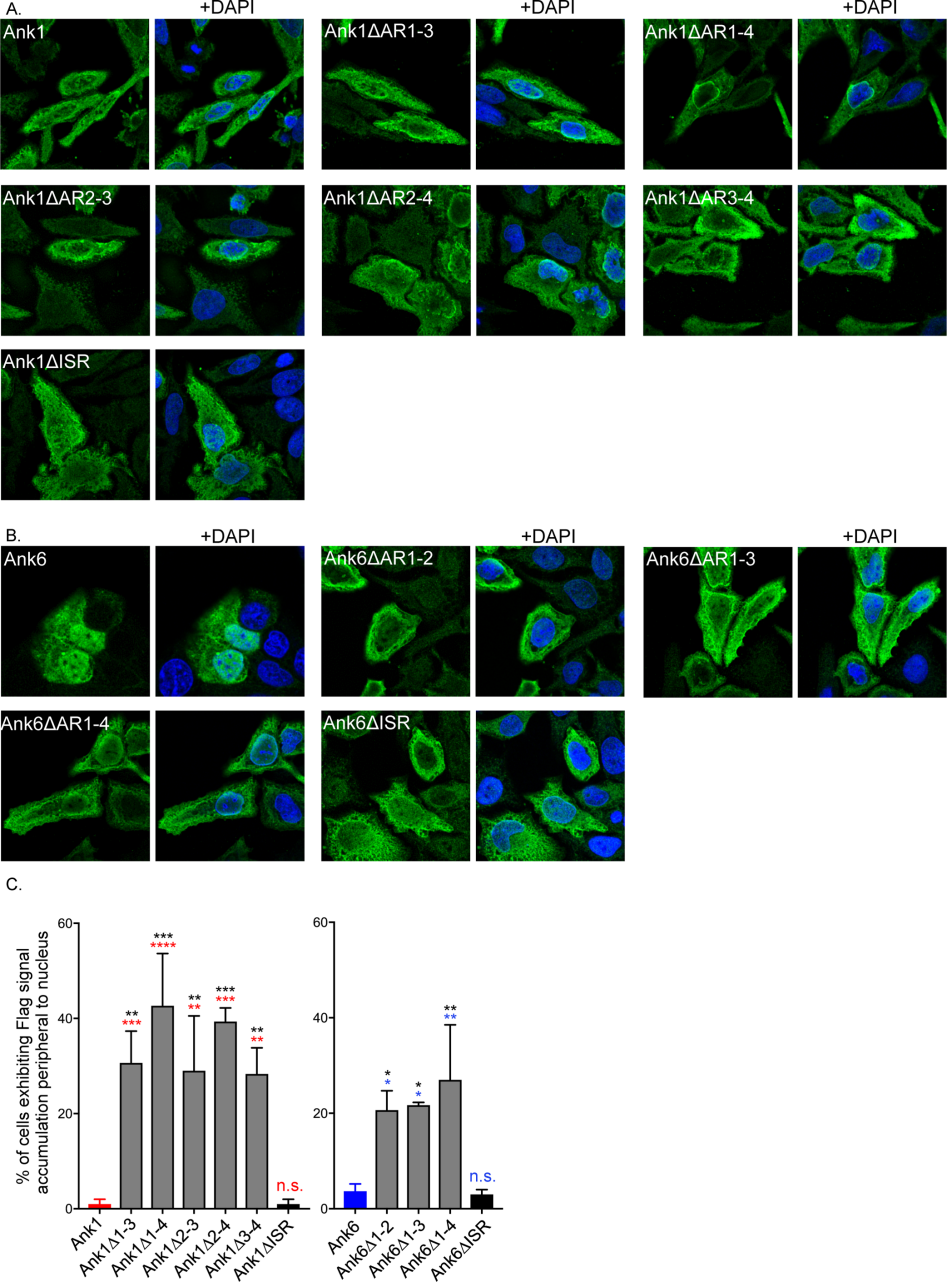
Identification of the Ank1 and Ank6 domains that are essential for optimal translocation into the nucleus. HeLa cells were transfected to express Flag-tagged versions of Ank1, Ank6, or the indicated deletion mutant thereof. At 16 h, the cells were fixed, screened with Flag tag antibody, stained with DAPI, and examined by confocal microscopy. (A and B) Representative fluorescence images of cells viewed for Flag-tagged Ank1 (A) or Ank6 proteins (B) with and without DAPI. (C) The mean percentage + SD of transfected cells exhibiting Flag signal accumulation around the periphery of nuclei was determined. Triplicate samples of 100 cells each were counted per condition. Data presented are indicative of three experiments with similar results. Statistically significant (*P<0.05; **P<0.01; ***P<0.001; ****P<0.0001) values are indicated. n.s., not significant. Indicators of statistical significance relative to values obtained for Flag-Ank1, Flag-Ank1△ISR, Flag-Ank6, and Flag-Ank6△ISR are colored red, black, blue, and black, respectively.

The ability of each Flag-tagged Ank1 or Ank6 deletion mutant to translocate into HeLa cell nuclei versus its wild-type counterpart was assessed using confocal microscopy. Comparable to Flag-Ank1, immunolabeled Flag-tagged Ank1 proteins lacking the N-terminus, ISR, F-box, any individual ankyrin repeat, ankyrin repeats one and two, ankyrin repeats one and four, and residues 143 to 160 were detected throughout the nuclei of transfected cells (Figs 16A, S8 and S2 Table). In contrast to the nucleotropic phenotype for which Flag-Ank1 and Flag-Ank1△ISR are representative, cells expressing Flag-tagged Ank1△AR1-3, Ank1△AR1-4, Ank1△AR2-3, Ank1△AR2-4, and Ank1△AR3-4 exhibited less distribution throughout the nuclei and instead concentrated around the periphery of nuclei (Fig 16A, 16C, and S2 Table). These data indicate that (1) the ankyrin repeat domain of Ank1 is essential for its ability to optimally translocate into the nucleus; (2) two tandemly-arranged ankyrin repeats can mediate this phenomenon provided that one of them is ankyrin repeat three; and (3) any three ankyrin repeats of Ank1 are sufficient to mediate optimal nuclear translocation. With the exception of Flag-Ank1△AR1-3, the Flag-Ank1 deletion mutants that were most defective in nuclear trafficking were also among the most compromised in binding importin β1 (Figs 15, 16A, 16C, S8, and S2 Table).

The pronounced nucleotropic phenotype exhibited by Flag-Ank6 was mimicked by Flag-tagged Ank6 proteins lacking the N-terminus, ankyrin repeat 3, or the F-box, indicating that, just as these three domains are expendable for optimally interacting with importin β1 (Fig 15), they are also dispensable for nuclear localization (Figs 16B, S8, and S2 Table). Deleting Ank6 ankyrin repeat one, two, or four or ankyrin repeats one and two, one through three, one through four, or two and three resulted in a redistribution of the protein from being primarily nuclear to primarily cytoplasmic. Additionally, Flag-Ank6△AR1-2, Flag-Ank6△AR1-3, and Flag-Ank6△AR1-4 concentrated around the periphery of the nucleus (Figs 16B, 16C, and S2 Table). Similar to that observed for Flag-Ank1 proteins, those Flag-Ank6 truncations that exhibited the nuclear ringing phenotype did so at a significantly greater incidence than Flag-Ank6 and Flag-Ank6△ISR (Figs 16B, 16C, and S2 Table). Flag-tagged Ank6△AR2-4, Ank6△AR3-4, and Ank6△1,4 exhibited an intermediate phenotype as they did not accumulate in nuclei as well as full-length Ank6 but did so more effectively than the other Ank6 truncation mutants that displayed nucleotropism defects (S8 Fig and S2 Table). The intermediate phenotype of these three truncated proteins is likely because, although optimal Ank6 nuclear translocation requires ankyrin repeats one, two, and four, having ankyrin repeat one alone, ankyrin repeats one and two, or ankyrin repeats two and three allow for some ectopically expressed protein to translocate into the nucleus, albeit less efficiently. Flag-Ank6△ISR and Flag-Ank6△NES also exhibited the intermediate nuclear localization phenotype (Figs 16B, S8, and S2 Table), the latter of which is likely because it lacks more than half of ankyrin repeat four. From these data, it can be extrapolated that Ank6 optimal nuclear trafficking involves ankyrin repeats one, two, and four and, of these three repeats, the first and second are the most contributory. The abilities of Flag-tagged Ank1△ISR and Ank6△ISR to enter the nucleus versus those of Ank1 or Ank6 lacking one or more ankyrin repeats confirms that altered nuclear trafficking exhibited by the ankyrin repeat truncations was specific and not simply due to deleting a portion of the protein. Overall, it can be concluded that distinct domains of Ank1 and Ank6 are essential for each to efficiently translocate into the nucleus and the same regions of these proteins that interact with importin β1 also mediate nuclear translocation.

### Ank1 and Ank6 F-box and ISR domains are essential for inhibiting p65 accumulation in the nucleus

**Fig 17:**
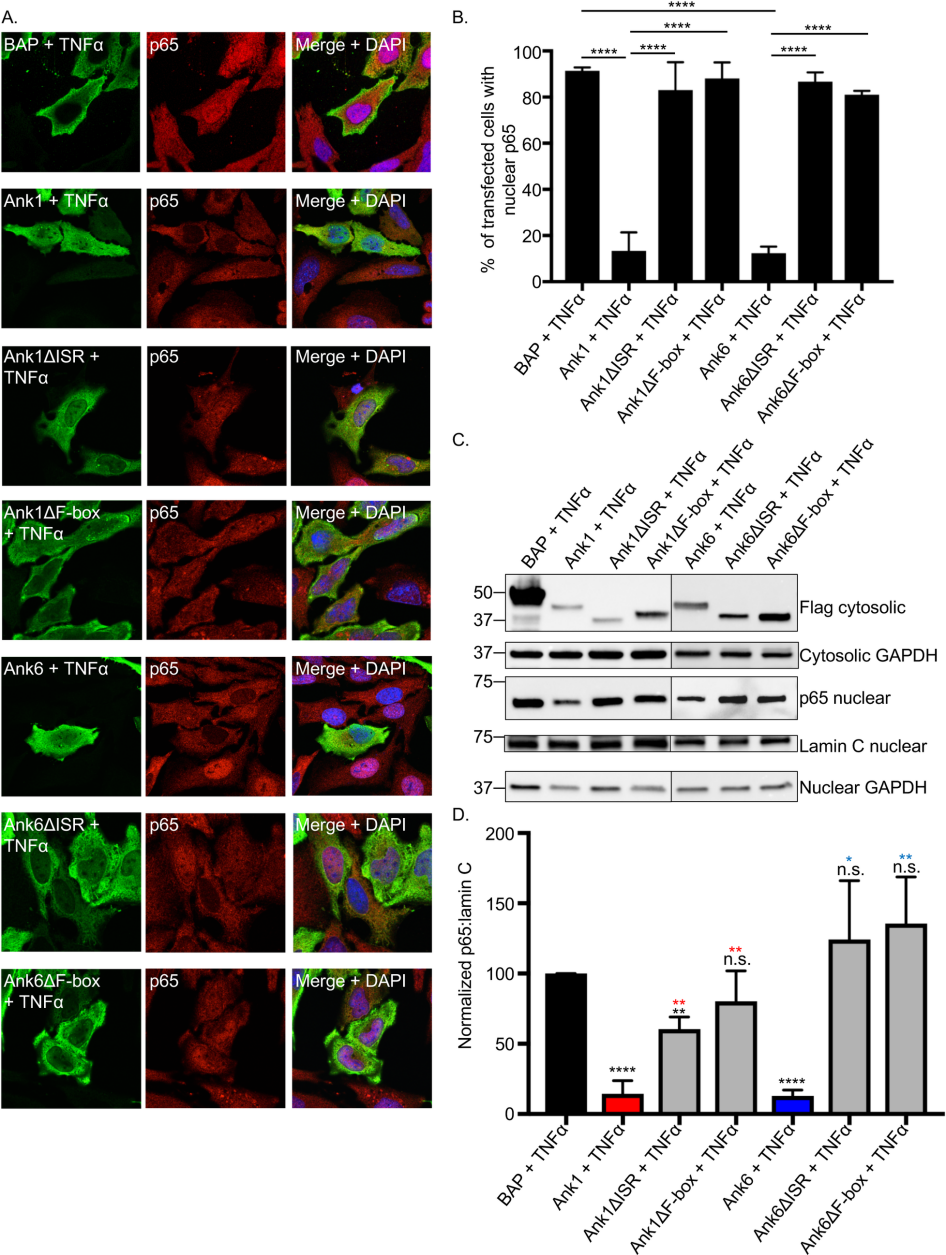
The ability of Ank1 and Ank6 to each optimally inhibit p65 nuclear accumulation requires the ISR and F-box domain. HeLa cells were transfected to express Flag-tagged BAP, Ank1, Ank1△ISR, Ank1△F-box, Ank6, Ank6△ISR, or Ank6△F-box. At 16 h, the cells were exposed to TNFα for 30 min, after which they either were fixed, screened with antibodies specific for the Flag epitope and p65, and examined by confocal microscopy (A and B) or resolved into nuclear and cytosolic fractions that were subjected to Western blot and densitometry analyses (C and D). (A) Representative fluorescence images of cells viewed for Flag-tagged protein, p65, and merged images plus DAPI. (B) The mean percentage + SD of transfected cells exhibiting p65 in the nucleus was determined. Triplicate samples of 100 cells each were counted per time point. (C) Western blotted nuclear and/or cytosolic fractions were screened with antibodies against the Flag epitope, GAPDH, p65, or lamin C. (D) Mean normalized ratios + SD of p65:lamin C densitometry signals were calculated from three separate experiments performed in (C). Results in each panel are representative of three independent experiments. Statistically significant (*P < 0.05; **P < 0.01; ****P<0.0001) values are indicated. n.s., not significant. Indicators of statistical significance relative to values obtained for Flag-BAP, Flag-Ank1, and Flag-Ank6 are colored black, red, and blue, respectively.

To identify the Ank1 and Ank6 domains that are necessary for inhibiting p65 nuclear accumulation, HeLa cells ectopically expressing Flag-tagged versions of each wild-type and deletion mutant protein and BAP were treated with TNFα for 30 min followed by examination by confocal microscopy. For both Ank1 and Ank6, deleting only the ISR or F-box ablated the ability to inhibit p65 accumulation in the nucleus (Fig 17A and 17B.). All other truncation mutants were unhindered in mediating this phenomenon (S9 and S10 Figs). To corroborate these findings, nuclear and cytosolic fractions of HeLa cells transfected to express Flag-tagged BAP, Ank1 or Ank6, or △ISR or △F-box versions thereof were treated with TNFα followed by Western blot and densitometry analyses. As previously observed (Fig 7A), the amount of p65 present in the nuclear fractions of cells expressing Flag-Ank1 or Flag-Ank6 post-TNFα stimulation was significantly reduced versus cells expressing Flag-BAP (Fig 17C and 17D). However, the amount of nuclear p65 in cells expressing Flag-tagged Ank1 or Ank6 lacking the ISR or F-box was significantly greater than that for their full-length counterpart and was comparable to that observed for cells expressing Flag-BAP. Overall, these data demonstrate that the ISR and F-box domains of Ank1 and Ank6 are essential for retarding p65 accumulation in the nucleus.

## Discussion

Within two h following entry into a host cell, *O*. *tsutsugamushi* exits the late endosome to the cytosol where it replicates for the remainder of its intracellular life cycle [65]. Our and others’ data indicate that host cell detection of the bacterium in the cytosol activates the NF-κB response [29, 66]. As NF-κB is essential for activating transcription of hundreds of antimicrobial response genes [27], it would be advantageous for *O*. *tsutsugamushi* to dampen NF-κB-mediated transcription. While this has been suggested [29, 66], confirmation of this phenomenon and exposition of the responsible mechanism have remained elusive. The p50/p65 NF-κB heterodimer is ubiquitously expressed in mammalian host cells [27]. In this report, we demonstrate that *O*. *tsutsugamushi* inhibits accumulation of p65 in the nuclei of immortalized human HeLa cells and primary murine BMDMs beginning within the first several h of infection. Inhibition of p65 is seen in cells infected at MOIs that are comparable to those observed for infected macrophages in eschar lesions that form at the chigger bite site [17]. *O*. *tsutsugamushi* promotes nuclear export of p65 in an exportin 1-dependent manner. Yet, the bacterium impairs NF-κB-activated transcription even when exportin 1 is functionally inhibited by LMB and NF-κB consequently remains in the nucleus. The ability of *O*. *tsutsugamushi* to modulate p65 presumably involves two Type I secretion system effectors, Ank1 and Ank6. Both are expressed during infection and, when ectopically expressed, inhibit NF-κB-dependent transcription by an exportin 1-independent means and antagonize p65 nuclear accumulation in an exportin 1-dependent manner to phenocopy events observed in *O*. *tsutsugamushi* infected cells. Flag-tagged Ank1 and Ank6 each interact with p65, importin β1, and exportin 1. Their abilities to translocate into the nucleus rely on interactions with importin β1 and are importazole-sensitive. Accordingly, we propose that *O*. *tsutsugamushi* Ank1 and Ank6 each interact with importin β1 to mobilize into the nucleus via the classical nuclear import pathway. Once intranuclear, they bind NF-κB to prevent it from activating transcription. NF-κB is subsequently shuttled out of the nucleus by exportin 1, either independently or as part of an Ank:NF-κB:exportin 1 ternary complex. Overall, this mechanism ultimately leads to reduction in nuclear levels of p65 and NF-κB-dependent expression of antimicrobial genes.

During the first 24 h of infection, *O*. *tsutsugamushi* growth is slow, but becomes logarithmic between 48 and 72 h [67]. The bacterium reduces p65 nuclear accumulation within the first 8 to 12 h, depending on the host cell type, indicating that it likely utilizes preformed effectors that target NF-κB and/or rapidly induces their expression upon host cell invasion. NF-κB can be activated by a variety of stimuli, including lipopolysaccharide (LPS) and TNFα [27]. *O*. *tsutsugamushi* lacks LPS, but induces TNFα expression during infection of human patients, experimentally infected mice, primary cells, and cultivated host cells [34, 38, 41, 68–72]. We therefore utilized TNFα as an external stimulus for NF-κB. *O*. *tsutsugamushi* could not effectively override TNFα-stimulated p65 nuclear accumulation at 24 h but was able to do so with increasing efficacy at 48 and 72 h, suggesting that this phenomenon might be bacterial dose-dependent. Indeed, NF-κB-activated luciferase expression in *O*. *tsutsugamushi* infected cells measured at 24 h was dampened in a bacterial load-dependent manner. Transiently overexpressed Ank1 and Ank6 each inhibit NF-κB activated transcription comparably to the IκBα SR, but not as effectively as *O*. *tsutsugamushi* itself. This could simply be due to the fact that not all cells expressed Ank1 or Ank6 following transient transfection versus >95% of cells being infected. Alternatively, the bacterium might utilize additional effectors that negatively regulate the NF-κB response.

Many pathogens inhibit the NF-κB response by degrading p65 or preventing either degradation of IκBα or its dissociation from NF-κB [27]. *O*. *tsutsugamushi* NF-κB modulation is distinct from these strategies. p65 levels are unchanged and IκBα levels are reduced to similar degrees in uninfected and infected cells upon TNFα stimulation. The ability of *O*. *tsutsugamushi* to inhibit TNFα-induced p65 nuclear accumulation is at least partially attributable to Ank1 and Ank6, as supported by studies of HeLa cells expressing Flag-tagged versions of these proteins. Similar to that observed for infected cells, p65 levels are unchanged in cells expressing Flag-Ank1 and Flag-Ank6. Ank1 and Ank6 are the only *O*. *tsutsugamushi* Anks that modulate the NF-κB response by preventing TNFα-induced accumulation of p65 in the nucleus. This inhibitory effect on p65 was not due to overexpression of a foreign protein, as p65 was pronouncedly observed in the nuclei of transfected cells expressing Flag-BAP or GFP following the addition of TNFα. Flag-tagged Ank1 and Ank6 precipitate p65, but not IκBα from lysates of TNFα-exposed HeLa cells. This suggests that TNFα-induced IκBα dissociation from p65 proceeds normally in the presence of either Ank and that a Flag-Ank1/6:p65:IκBα ternary complex does not form. Ank1 and Ank6 also precipitate p65 but not IκBα in the absence of TNFα, suggesting that they each might be able to outcompete IκBα for binding to p65. While the possibility that one or more unidentified *O*. *tsutsugamushi* effectors modulate IκBα cannot be absolutely excluded, the data presented herein argue against this possibility.

There are at least six different importin α proteins, most of which interact with importin β1 to facilitate NF-κB nuclear transport [46]. It is unknown if Ank1 or Ank6 directly interact with importin α1 or indirectly via binding to an importin α protein. Some eukaryotic nuclear trafficking proteins can interact directly with importin β1 in the absence of an importin α protein to facilitate their nuclear import [73, 74]. However, if Ank1 and Ank6 were to directly bind importin α, it is unclear how they would do so because both lack a canonical NLS. Additionally, although Ank1 and Ank6 interact with p65, they likely exclusively traffic to the nucleus via importin β1 as opposed to directly hitching a ride on p65 as it translocates into the nucleus. We conclude this because Flag-tagged versions of both effectors accumulate in the nucleus in the absence of TNFα, which would promote p65 import into the nucleus.

Other microbial proteins co-opt either an exportin or importin protein but not both, and none of these examples directly impact NF-κB function. Hantaan virus nucleocapsid protein, which binds importin α1, importin α2, and importin α3 but neither p50 nor p65, is believed to inhibit NF-κB nuclear import by sequestering the importins from interacting with p50:p65 [75]. *Salmonella enterica* Typhimurium SpvD interacts with exportin Xpo2, which mediates nuclear-cytosolic recycling of importins. This interaction has been proposed to disrupt the normal recycling of importin α from the nucleus to thereby indirectly inhibit p65 nuclear translocation [76]. African swine fever virus protein, A238L, which inhibits NF-κB activation, translocates out of the nucleus via the exportin 1 pathway but does not promote exportin 1-dependent p65 nuclear export [77]. *Yersinia enterocolitica* YopM, which translocates to the nucleus but does not target NF-κB, interacts with DDX3 to exit the nucleus via the exportin 1 pathway [78]. To our knowledge, Ank1 and Ank6 are the first examples of microbial proteins that exploit both importin and exportin activities and do so to dysregulate NF-κB.

Ank1 and Ank6 co-immunoprecipitation of p65, importin β1, and exportin 1 are specific and not simply due to their having an ankyrin repeat domain. Neither Flag-BAP nor Flag-Ank7 co-immunoprecipitate any of the three Ank1 and Ank6 interacting partners. Nearly all *O*. *tsutsugamushi* Anks carry a C-terminal F-box domain that is capable of binding SKP1 and nucleating the SCF1 complex, including Ank1 and Ank6 [22, 23]. Thus, these two Anks can form interactions with multiple host cell proteins enabling them to exploit/modulate nuclear import and export pathways, NF-κB transcriptional activation, and SCF1 complex assembly. We recently reported the ability of *O*. *tsutsugamushi* Ank9 to manipulate Golgi-to-ER retrograde trafficking, the unfolded protein response, protein secretion, and SCF1 complex assembly [24]. Collectively, Ank1, Ank6, and Ank9 evidence an emerging theme in *O*. *tsutsugamushi* pathobiology by which individual Ank effectors can exploit multiple host cell pathways. This arguably enables it to maximize use of its small genome derived from its reductive evolution as an obligate intracellular bacterium.

Though Ank1 and Ank6 are functionally identical in terms of how they modulate NF-κB, they bear only 58.6% overall amino acid identity. Even more striking, the greatest disparity in their sequence similarity occurs within the ankyrin repeat region, which is one of the most common protein-protein interaction motifs in nature [21] and is essential for interacting with p65, importin β1, and exportin 1. Ankyrin repeat domains are tandemly-arrayed helix-turn-helix-loop motifs that bind their ligands with specific residues in the loops and on the surface of the helical array proximal to the loops [79, 80]. Key Ank1 and Ank6 residues in individual ankyrin repeats and/or combinations thereof are likely responsible for binding the host cell interacting partners. Each ankyrin repeat and ISR of Ank1 and Ank6 contributes to binding p65. The Ank1 and Ank6 truncation mutant binding profiles for p65 and exportin 1 are similar, suggesting that the effectors might not directly bind exportin 1 but do so indirectly through p65. From the co-immunoprecipitation data, it could not be discerned whether the Ank6 putative NES contributes to the ability to interact with exportin 1. If it does, this region alone is insufficient.

The F-box and ISR are the only two domains tested that are essential for Ank1 and Ank6 to inhibit p65 accumulation in the nucleus. Ectromelia virus encodes a series of four F-box-containing Anks that inhibit p65 nuclear translocation in an F-box dependent manner, though they do so by inhibiting TNFα- and IL-1β-induced IκBα degradation [60]. Ank1 and Ank6 are the first examples of bacterial F-box domains that negatively regulate NF-κB. Because TNFα-stimulated IκBα degradation proceeds normally in *O*. *tsutsugamushi* infected cells, it can be inferred that the Ank1/Ank6 mechanism of action is distinct from the ectromelia virus F-box-containing Anks. Given the ability of the F-box to nucleate ubiquitin ligase complexes [22, 23], these effectors might antagonize NF-κB function by mediating ubiquitination of it or another interacting partner. It was recently discovered that monoubiquitination of p65 decreases NF-κB transcriptional activity independent of proteasomal degradation even when NF-κB is retained in the nucleus for a prolonged period; and this has been linked to the inability of NF-κB to interact with its transcriptional co-activator, CREB-binding protein [81]. In line with these findings, it was demonstrated herein that *O*. *tsutsugamushi* or Flag-tagged Ank1 or Ank6 inhibits NF-κB-mediated transcriptional activation and that p65 levels are not reduced in cells expressing Flag-Ank1 or Flag-Ank6. Based on studies of other microbial F-box proteins, including the recent elucidation of the co-crystal of *L*. *pneumophila* AnkB and human SKP1 [82], the prevailing model for this class of effectors is that the ankyrin repeat domain binds a specific host cell target protein and the F-box recruits SKP1 to nucleate a ubiquitin ligase complex that ubiquitinates the bound protein. In this context, a presumptive role for the ISR could be to link these two functional domains, though co-immunoprecipitation studies presented herein indicate that the ISR also contributes to protein-protein interactions. Moving forward, dissecting the specific mechanism(s) by which the F-box and ISR of Ank1 and Ank6 negatively regulate NF-κB function will be of paramount importance.

Uncontrolled NF-κB activation can lead to inflammation, autoimmune diseases, and cancer, making it an attractive target for treating these disorders [27]. With millions of years of evolution as a head start, intracellular microbes have already devised strategies to modulate the NF-κB pathway. This report revealed that *O*. *tsutsugamushi* modulates NF-κB p65 nuclear transport via the unprecedented actions of two Ank effectors, thereby advancing understanding of *O*. *tsutsugamushi* pathobiology and expanding the repertoire of mechanisms by which microbes modulate the antimicrobial response. It also underscores how ankyrin repeats enable individual effectors to interface with multiple target proteins to co-opt diverse host cell processes and alludes to a previously unrecognized means by which the F-box domain negative regulates NF-κB. Further study of Ank1 and Ank6 will continue to generate information that could be exploited to therapeutically manipulate NF-κB.

## Materials and methods

### Ethics statement

This study was conducted in strict accordance with the recommendations in the Guide for the Care and Use of Laboratory Animals of the National Institutes of Health. Mouse experiments (protocol AM10220) were approved by the Institutional Animal Care and Use Committee, Virginia Commonwealth University Animal Welfare Assurance Number A2381-01. Mice were euthanized by carbon dioxide inhalation followed by exsanguination.

### Cultivation of cell lines and *O*. *tsutsugamushi*

HeLa human cervical epithelial cells (CCL-2; American Type Culture Collection [ATCC], Manassas, VA) were cultured in Roswell Park Memorial Institute (RPMI) 1640 medium supplemented with 10% fetal bovine serum (FBS; Gemini Bio-Products, Sacramento, CA, USA). HeLa cells stably transfected to contain four copies of the NF-κB response element upstream of the Firefly Luciferase gene (Signosis, Santa Clara, CA) were maintained in Dulbecco’s modified Eagle’s medium (DMEM) with L-Glutamine, 4.5 g l^−1^ D-Glucose, 100 mg l^−1^ sodium pyruvate (Thermo Fisher Scientific, Waltham, MA), and supplemented with 10% FBS. L929 mouse fibroblast cells (CCL-1 NTC Clone 929; ATCC) were cultured in Earle’s balanced salts with L-glutamine minimum essential medium (EMEM; Quality Biological, Gaithersburg, MD) with 2.5% FBS to create L929 conditioned media. Uninfected and *O*. *tsutsugamushi* infected RAW264.7 cells (TIB-71; ATCC) were cultivated in DMEM with 10% FBS. Bone marrow-derived macrophages (BMDMs) were generated by cultivating bone marrow cells obtained from the femurs of 6- to 8-week old C57BL/6 mice (Jackson Laboratory; Bar Harbor, ME) in DMEM containing 30% L929 conditioned media, 3.3% FBS, and 1X Anti-Anti (Thermo Fisher Scientific) (BMDM differentiating medium) at 37°C in 5% CO_2_ for 5 or 6 days. The BMDM differentiating medium was changed every 3 days. Macrophage differentiation was confirmed by direct immunofluorescent microscopy using fluorescein isothiocyanate-conjugated CD11b antibody (Biolegend, San Diego, CA). *O*. *tsutsugamushi* str. Ikeda was originally isolated from a patient in Japan [83]. *O*. *tsutsugamushi* str. Ikeda was maintained in HeLa cells as described for uninfected cells except that it had 1X Anti-Anti and the FBS concentration was 1%, the latter of which slowed host cell growth and thereby allowed for a high percentage (>90%) of infection to be achieved after 3 to 4 days. To obtain *O*. *tsutsugamushi* for experimental use, infected HeLa cells were mechanically disrupted using glass beads followed by differential centrifugation at 200 x *g* for 4 min to remove intact cells and cellular debris. The resulting supernatant was centrifuged at 2,739 x *g* for 10 min to obtain *O*. *tsutsugamushi* organisms for use in infection studies. In infection experiments, *O*. *tsutsugamushi* infected and uninfected control cells were maintained in the appropriate media containing 10% FBS.

### Plasmid constructs

pFLAG-Ank01_02 and pFLAG-Ank06_02 for expressing Flag-tagged mammalian codon-optimized Ank1 and Ank6, respectively, have been described previously (Viebrock *et al*., 2015). To generate pFLAG-p65, cDNA was generated from HL-60 cells (CCL-240; ATCC) as described (Troese *et al*., 2011). Human p65 was PCR amplified from the cDNA template using primers 5’-**ATAGATCTGATATC**GGTACCAATGGACGAACTGTTCCCC-3’ and 5’-**AGTCAGCCCG**GGATCCGGAGCTGATCTGACTCAGCAG-3’ (boldface indicates nucleotides that are complementary to the sequences in the p3XFLAG-CMV-7.1 plasmid [Sigma-Aldrich, St. Louis, MO]; KpnI and BamHI restriction sites are underlined) as described (VieBrock *et al*., 2015). The amplicon was ligated into p3XFLAG-CMV-7.1 (Sigma-Aldrich, St. Louis, MO) plasmid that had been digested with KpnI and BamHI by InFusion (Clontech, Mountain View, CA) cloning as described [25]. Plasmid encoding Flag-tagged IκBα super repressor (pFLAG-IκBα SR), which contains the human IκBα gene having alanine substitutions at serine 32 and serine 36 to prevent its phosphorylation and thus its degradation (Brockman *et al*., 1995), was a kind gift from Dean W. Ballard (Vanderbilt University, Nashville, TN) and Tomasz Kordula (Virginia Commonwealth University, Richmond, VA). pFLAG-BAP, which encodes Flag-tagged BAP was purchased from Sigma-Aldrich. pBMC vectors carrying insert sequences encoding mammalian codon-optimized Ank1△AR2, Ank1△AR3, Ank1△AR2-3, Ank6△AR2, Ank6△AR3, and Ank6△AR2-3 were synthesized by Genewiz (South Plainfield, NJ). Constructs encoding Flag-tagged Ank1 and Ank6 deletion mutants listed in Fig 14 were generated using pFLAG-Ank01_02 or pFLAG-Ank06_02 [25] or pBMC-Ank1△AR2, pBMC-Ank1△AR3, pBMC-Ank1△AR2-3, pBMC-Ank6△AR2, pBMC-Ank6△AR3, or pBMC-Ank6△AR2-3 as template and primers listed in S3 and S4 Table. Amplicons were cloned into p3XFLAG-CMV-7.1 using either the InFusion (Clontech) approach or ligation-independent cloning as described [24, 25].

### NF-κB and Flag-tagged protein nuclear translocation immunofluorescence assay

HeLa cells were seeded onto glass coverslips in 24-well plates. For infection experiments, cells were infected with *O*. *tsutsugamushi* and incubated at 35°C. For mock infected controls, uninfected HeLa cells were mechanically disrupted and differentially centrifuged exactly as described above for *O*. *tsutsugamushi* infected cells. For the mock infection control, the resulting miniscule pellet of uninfected HeLa cellular debris was resuspended in RPMI 1640 medium containing 10% (vol/vol) FBS and the suspension was incubated with naïve HeLa cells for the same period of time as host cell free bacteria. For studies involving ectopic expression of Flag-tagged proteins, HeLa cells were transfected with 0.4 µg of plasmid using Lipofectamine 2000 (Invitrogen, Carlsbad, CA) as directed by manufacturer. Cells were allowed to ectopically express protein for 16 h before being processed. In some cases, cells were incubated with media containing 25 ng/mL of TNFα (Life Technologies, Grand Island, NY) or vehicle control (H_2_O) for 30 min at 37°C. Cells were then fixed and permeabilized with -20°C methanol. The coverslips were washed with phosphate buffered saline (PBS; 1.05 mM KH_2_PO_4_, 155 mM NaCl, 2.96 mM Na_2_HPO_4_, pH 7.4) twice and then blocked in 5% (vol/vol) bovine serum albumin (BSA) in PBS for 1 h at room temperature. Samples were then incubated at room temperature with antibody targeting endogenous p65 at a 1:250 (Santa Cruz, Dallas, TX) or 1:1000 (Invitrogen) dilution, rabbit anti-*O*. *tsutsugamushi* TSA56 at a 1:1,000 dilution [24], and/or mouse anti-Flag epitope (Sigma-Aldrich) at a 1:1,000 dilution in PBS containing 5% (vol/vol) BSA for 1 h. The coverslips were washed three times in PBS and incubated with Alexa Fluor 488-conjugated goat anti-mouse or anti-rabbit IgG (Invitrogen) and Alexa Fluor 594-conjugated goat anti-mouse or anti-rabbit IgG (Invitrogen) at a 1:1,000 dilution in 5% BSA for 1 h at room temperature. Samples were incubated with 0.1 µg ml^-1^ 4’ 6-diamidino-2-phenylindole (DAPI; Invitrogen) in PBS for 1 min, rinsed with PBS three times, and mounted using ProLong Gold Antifade mounting media (Invitrogen). Coverslips were imaged with a Zeiss LSM 700 laser-scanning confocal microscope in the Virginia Commonwealth University Department of Anatomy and Neurobiology Microscopy Facility. Cells were scored for NF-κB nuclear accumulation by counting 100 cells per coverslip in a blinded fashion, examining for the presence of immunolabeled p65 in the cytosol or nucleus. To inhibit nuclear import and export, respectively, cells were treated with 50 µM importazole (Sigma-Aldrich) for 3 h or 20 nM LMB (Sigma-Aldrich) for 1 h prior to processing for immunofluorescence microscopy.

### NF-κB luciferase assay

HeLa NF-κB luciferase cells were seeded at a density of 5 x 10^4^ cells per well in a 96-well plate and cultivated overnight. For infection studies, cells were infected with *O*. *tsutsugamushi* at an MOI of 10, 50, or 100 and incubated at 35°C for 24 h. MOIs were confirmed by immunofluorescent microscopy examination of cells that had been infected in parallel. In some cases, the HeLa NF-κB luciferase cells were transfected with 0.1 µg of plasmid to express Flag-tagged protein for 16 h prior to being assayed. The media was replaced with fresh media containing 25 ng/mL TNFα or vehicle control and incubated for 8 h for infection studies or 4 h for ectopic expression studies. The media was removed and the cells were rinsed with 100 µL of PBS followed by a 15-min incubation in 20 µL of Passive Lysis buffer (Promega, Durham, NC). The samples were then vigorously pipetted with 100 µL of Firefly Luciferase substrate (Signosis). Luminescence signal was quantitated using a Perkin Elmer 1420 Multilabel Counter Victor3 for 10 s per well. To determine if exportin 1 activity is critical for *O*. *tsutsugamushi* to inhibit TNFα -stimulated NF-κB-dependent transcriptional activation, HeLa NF-κB luciferase cells were infected at an MOI of 10 for 24 h. The cells were then treated with 5 nM LMB in 70% vol/vol methanol or vehicle control together with TNFα (25 ng/ml) for 8 h and the assay continued as described above. To confirm whether exportin 1 activity is important for the ability of Flag-tagged Ank1 or Ank6 to inhibit TNFα-stimulated NF-κB-dependent transcriptional activation, HeLa NF-κB luciferase cells were transfected to express either Flag-Ank protein or control protein. At 16 h, the cells were then treated with 5 nM LMB in 70% vol/vol methanol or vehicle control together with TNFα (25 ng/ml) for four h and the assay continued.

### Western blotting

SDS-PAGE and Western blotting were performed as previously described [25]. Blots were probed with antibodies in tris-buffered saline with Tween-20 (TBS-T; 25 mM Tris HCl, 137 mM NaCl, 2.7 mM KCl, 0.05% Tween-20; pH 7.4) containing 5% (vol/vol) non-fat milk. Western blot screening was done using mouse/rabbit anti-Flag (Sigma-Aldrich) at a 1:1,000 dilution, rabbit anti-p65 (Santa Cruz) at a 1:250 dilution, rabbit anti-p65 (Thermo Fisher Scientific) at a 1:250 dilution, rabbit anti-IκBα (Santa Cruz) at a 1:250 dilution, mouse anti-GAPDH (Santa Cruz) at a 1:250 dilution, mouse anti-β-actin (Santa Cruz) at a 1:2,500 dilution, mouse anti-lamin B1 (Santa Cruz) at a 1:250 dilution, rabbit anti-lamin A/C (Santa Cruz) at a 1:1,000 dilution, rabbit anti-importin β1 (Santa Cruz) at a 1:250 dilution, rabbit anti-exportin 1 (EMD Millipore, Billerica, MA) at a 1:10,000 dilution, and rabbit anti-*O*. *tsutsugamushi* TSA56 [24] at a 1:1,000 dilution. Bound primary antibodies were detected using horseradish-peroxidase-conjugated horse anti-mouse or anti-rabbit IgG (Cell Signaling Technology, Danvers, MA) at a 1:10,000 dilution in TBS-T containing 5% (vol/vol) milk. All blots were incubated with SuperSignal West Dura or SuperSignal West Femto chemiluminescent substrate (Thermo Fisher Scientific) and imaged using a ChemiDoc Touch Imaging System (Bio-Rad, Hercules, CA). Densitometry analysis was performed using Bio-Rad Image Lab software.

### Cytosol and nuclear fractionation

HeLa cells were grown in a T-75 flask to 60% confluency, which corresponds to approximately 5 x 10^6^ cells, for infection studies and to 90% confluency, which corresponds to approximately 7.5 x 10^6^ cells, for ectopic protein expression studies. For infection experiments, cells were incubated with *O*. *tsutsugamushi* at an MOI of 50 or with uninfected HeLa cell lysate for the mock infection control and incubated for 4 or 72 h. For protein expression studies, cells were transfected with 18 µg of plasmid DNA using Lipofectamine 2000 (Invitrogen) and incubated for 16 h. Old media was removed and replaced with fresh media containing TNFα (25 ng/ mL) or H_2_O as a vehicle control and incubated for 30 min. Media was removed from the flasks. The cells were rinsed with PBS, treated with 0.05% trypsin-EDTA (Invitrogen) for 5 min. Cells were harvested by centrifugation at 200 x g for 4 min, washed with PBS, and subjected to a second round of centrifugation. Cytosol and nuclear fractions were acquired using the Nuclear Extraction Kit (Abcam) and subjected to Western blot analysis.

### Immunoprecipitation

HeLa cells were transfected as described above to express Flag-taged proteins and incubated for 16 h. Cells were harvested and lysed in Tris buffer high saline (50 mM Tris HCl, 400 mM NaCl, 1 mM EDTA, pH 7.4) with 1.0% Triton x-100 (TBHS-T) containing protease inhibitor cocktail (Roche Diagnostics GmBH, Mannheim, Germany) on ice for 30 min with 10 s vortexing every 5 min. Agarose beads conjugated to mouse anti-Flag or rabbit anti-p65 antibodies were washed with TBHS-T buffer five times, centrifuged at 8,400 x g for 30 s, and added to 400 µg of cell lysate in final volume 500 µl. The samples were rotated with beads at 4°C overnight or room temperature for 4 h followed by centrifugation at 8,400 x g for 30 s and washing with TBHS-T five times. Beads were then resuspended in 30 µL of 2X Laemmli buffer and incubated at 100°C for 5 min to elute bound proteins. Inputs (50 µg of lysate) and eluates (entire 30 µL sample) were resolved by SDS-PAGE and screened by Western blotting.

### Statistical analysis

The Student’s t-test or one-way analysis of variance (ANOVA) was performed using the Prism 5.0 software package (Graphpad, San Diego, CA). Statistical significance was set to *P* < 0.05.

## Supporting information

S1 Fig HeLa cells infected with *O*. *tsutsugamushi* at a MOI as high as 100 remain intact for up to 72 h. HeLa cells were infected with *O*. *tsutsugamushi* at an MOI of 10, 25, 50, or 100. At 4, 24, or 72 h, the cells were fixed and screened with antibodies against *O*. *tsutsugamushi* TSA56 (*Ot*) and p65 prior to examination by confocal microscopy. Representative fluorescence images of cells viewed for *Ot*, p65, and merged images plus DAPI are presented. Results are representative of three independent experiments.
(TIF)

S2 Fig BMDMs infected with *O*. *tsutsugamushi* at a MOI as high as 100 remain intact for up to 72 h. BMDMs were infected with *O*. *tsutsugamushi* at an MOI of 10, 25, 50, or 100. At 4, 24, or 72 h, the cells were fixed and screened with antibodies against *O*. *tsutsugamushi* TSA56 (*Ot*) and p65 prior to examination by confocal microscopy. Representative fluorescence images of cells viewed for *Ot*, p65, and merged images plus DAPI are presented. Results are representative of three independent experiments.
(TIF)

S3 Fig Ank1 and Ank6, but no other Ank translocate into the nucleus and prevent p65 nuclear accumulation. HeLa cells were transfected to express Flag-BAP; GFP; Flag-tagged Ank1, Ank2, Ank3, Ank4, Ank5, Ank6, Ank7, Ank8, Ank9, Ank11, Ank12; or GFP-Ank10. At 16 h, the cells were exposed to TNFα or vehicle control for 30 min, after which they were fixed, screened with antibodies specific for p65 and the Flag epitope or GFP and examined by confocal microscopy. Images of cells expressing a given recombinant Ank treated with vehicle control are not presented because in all such cases p65 was absent from the nucleus and looked exactly like the panels presented for BAP- or GFP-expressing cells not treated with TNFα. Results are representative of three independent experiments.
(TIF)

S4 Fig Ank13 through Ank20 fail to translocate into the nucleus and prevent p65 nuclear accumulation. These data pair with data presented in S3 Fig. HeLa cells were transfected to express Flag-tagged Ank13, Ank15, Ank16, Ank17, Ank18, Ank19, Ank20, or GFP-Ank14. At 16 h, the cells were exposed to TNFα or vehicle control for 30 min, after which they were fixed, screened with antibodies specific for p65 and the Flag epitope or GFP and examined by confocal microscopy. Results are representative of three independent experiments.
(TIF)

S5 Fig Alignment of Ank1 and Ank6. Amino acid positions are listed to the left of the sequences. The arrows labeled AR (ankyrin repeat) 1 through 4 correspond to the individual ankyrin repeats. The AR, ISR, and PRANC domains are shaded blue, purple, and orange, respectively. The non-shaded region of Ank1 (residues 122 to 157) that aligns with Ank6 AR4 (residues 122 to 158) bears 41.7% identity with AR4, but is not annotated as an AR. Because eukaryotic ankyrin repeat domains can facilitate nuclear localization through RanGDP binding at two consecutive ankyrin repeats provided that a hydrophobic residue is located at the thirteenth position in each of the two repeats, the thirteenth residue of each AR, including the potential fourth AR of Ank1, is boxed. Hydrophobic amino acids at this position are indicated by red boldface text. The putative NES of Ank6 at amino acids 143 to 161 is underlined. Residues that make up the F-box are denoted by black boldface text. Amino acids that are identical between Ank1 and Ank6 are underscored by asterisks.
(TIF)

S6 Fig Importazole exhibits a modest effect on cytosolic levels of ectopically expressed Flag-tagged proteins. HeLa cells were transfected to express Flag-tagged Ank1, Ank6, or Ank9. At 16 h, the cells were treated with importazole or vehicle for 3 h. Following lysis and nuclear fractionation, Western blotted nuclear or cytosolic fractions were screened with antibodies against the Flag epitope, lamin A/C, and GAPDH. Mean ratios + SD of Flag:GAPDH densitometric signals from three separate Western blots, a representative image of which is presented in Fig 10A, were normalized to that for Flag-Ank9 to determine the effect of importazole on cytosolic levels of the ectopically expressed Flag-tagged Anks. Statistically significant (**P < 0.01) values are indicated. n.s., not significant. Data presented are representative of three independent experiments.
(TIF)

S7 Fig Ank1 and Ank6 remove p65 from the nucleus in an exportin 1-dependent manner. HeLa cells were transfected to express Flag tagged BAP, Ank1, Ank6, or IκBα SR. At 16 h, the cells were treated with LMB or vehicle control for 1 h. The media was replaced with media containing TNFα or vehicle for 30 min. The cells were then fixed, screened with antibodies specific for the Flag epitope and p65, and examined by confocal microscopy. Representative fluorescence images of cells viewed for Flag signal, p65, and merged images plus DAPI are presented. Results are representative of three independent experiments.
(TIF)

S8 Fig Ank1 and Ank6 domains that are dispensable for optimal translocation into the nucleus. HeLa cells were transfected to express the indicated Flag-tagged deletion mutants of Ank1 or Ank6. At 16 h, the cells were fixed, screened with Flag tag antibody, stained with DAPI, and examined by confocal microscopy. Representative fluorescence images of cells viewed for Flag-tagged Ank1 (A) and Ank6 proteins (B) with and without DAPI are presented. Triplicate samples of 100 cells were counted per condition. Data presented are indicative of three experiments with similar results.
(TIF)

S9 Fig The N-terminal region and ankyrin repeat domains of Ank1 do not contribute to its ability to inhibit p65 accumulation in the nucleus. HeLa cells were transfected to express Flag-tagged BAP or the indicated Ank1 deletion mutants. At 16 h, the cells were exposed to TNFα for 30 min after which they were fixed, screened with antibodies specific for the Flag epitope and p65, and examined by confocal microscopy. Representative fluorescence images of cells viewed for Flag-tagged protein, p65, and merged images plus DAPI are presented. Data obtained for cells expressing Flag-tagged Ank1, Ank1△ISR, and Ank1△F-box, the latter two of which are compromised in the ability to inhibit p65 nuclear accumulation, are presented in Fig 17.
(TIF)

S10 Fig The N-terminal region and ankyrin repeat domains of Ank6 do not contribute to its ability to inhibit p65 accumulation in the nucleus. HeLa cells were transfected to express Flag-tagged BAP or the indicated Ank6 deletion mutant. At 16 h, the cells were exposed to TNFα for 30 min after which they either were fixed, screened with antibodies specific for the Flag epitope and p65, and examined by confocal microscopy. Representative fluorescence images of cells viewed for Flag-tagged protein, p65, and merged images plus DAPI are presented. Data obtained for cells expressing Flag-tagged Ank6, Ank6△ISR, and Ank6△F-box, the latter two of which are compromised in the ability to inhibit p65 nuclear accumulation, are presented in Fig 17.
(TIF)

S1 Table Amino acid similarities between regions of *O*. *tsutsugamushi* str. Ikeda Ank1 and Ank6. (PDF)

S2 Table Correlation of Flag-Ank1 and Flag-Ank6 abilities to interact with importin β1 and translocate into the nucleus. (PDF)

S3 Table Oligonucleotide primers used in this study. (PDF)

S4 Table Primers utilized for InFusion generation of constructs encoding truncated Anks. (PDF)
