# Supplementary figures and images for: Orientia tsutsugamushi uses two Ank effectors to modulate NF-κB p65 nuclear transport and inhibit NF-κB transcriptional activation
Source: PLoS Pathog. 2018 May 7;14(5):e1007023. doi: 10.1371/journal.ppat.1007023 (PMC5957444; doi:10.1371/journal.ppat.1007023)

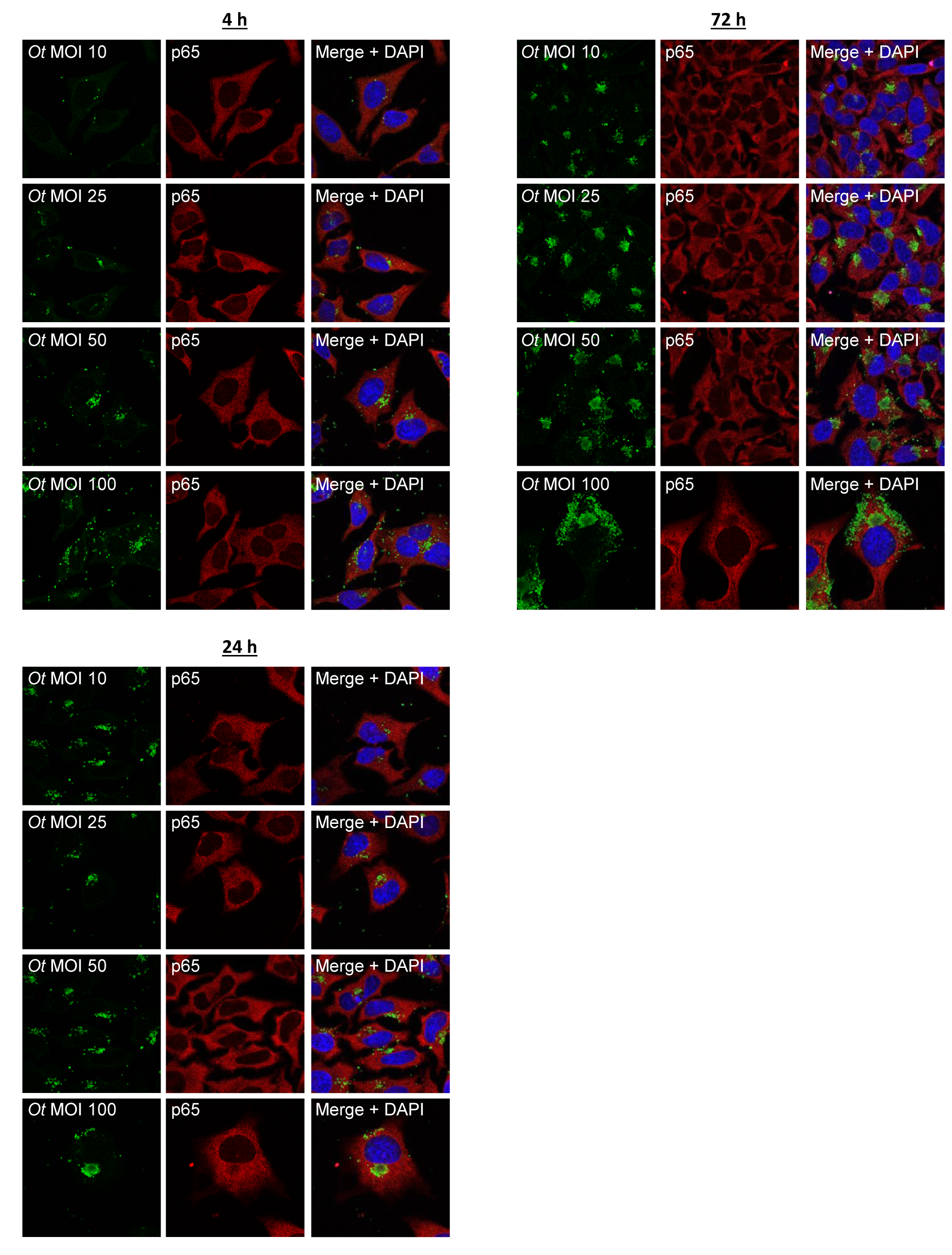

Supplement: S1 Fig — HeLa cells were infected with O. tsutsugamushi at an MOI of 10, 25, 50, or 100. At 4, 24, or 72 h, the cells were fixed and screened with antibodies against O. tsutsugamushi TSA56 (Ot) and p65 prior to examination by confocal microscopy. Representative fluorescence images of cells viewed for Ot, p65, and merged images plus DAPI are presented. Results are representative of three independent experiments. (TIF) [file ppat.1007023.s001.tif]

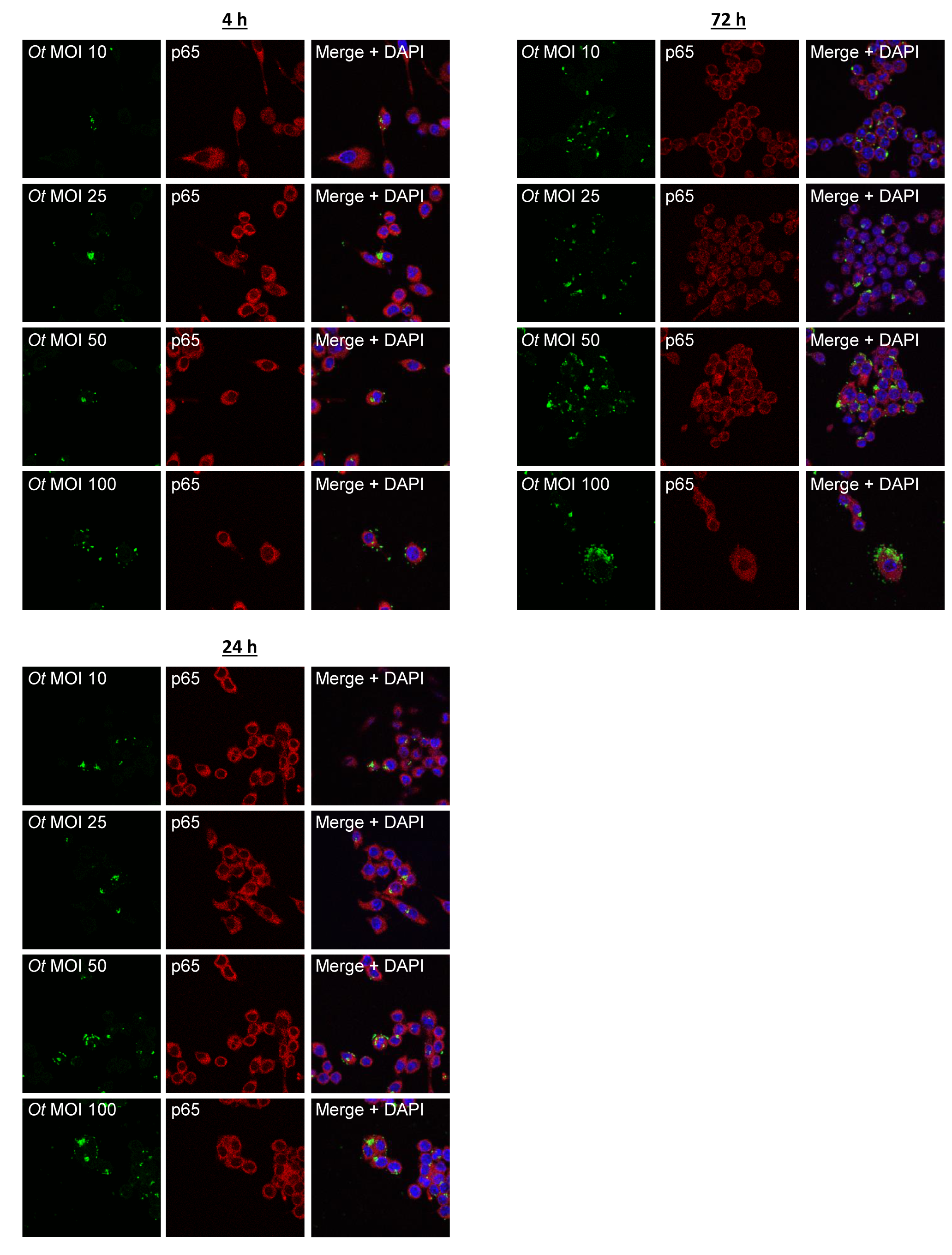

Supplement: S2 Fig — BMDMs were infected with O. tsutsugamushi at an MOI of 10, 25, 50, or 100. At 4, 24, or 72 h, the cells were fixed and screened with antibodies against O. tsutsugamushi TSA56 (Ot) and p65 prior to examination by confocal microscopy. Representative fluorescence images of cells viewed for Ot, p65, and merged images plus DAPI are presented. Results are representative of three independent experiments. (TIF) [file ppat.1007023.s002.tif]

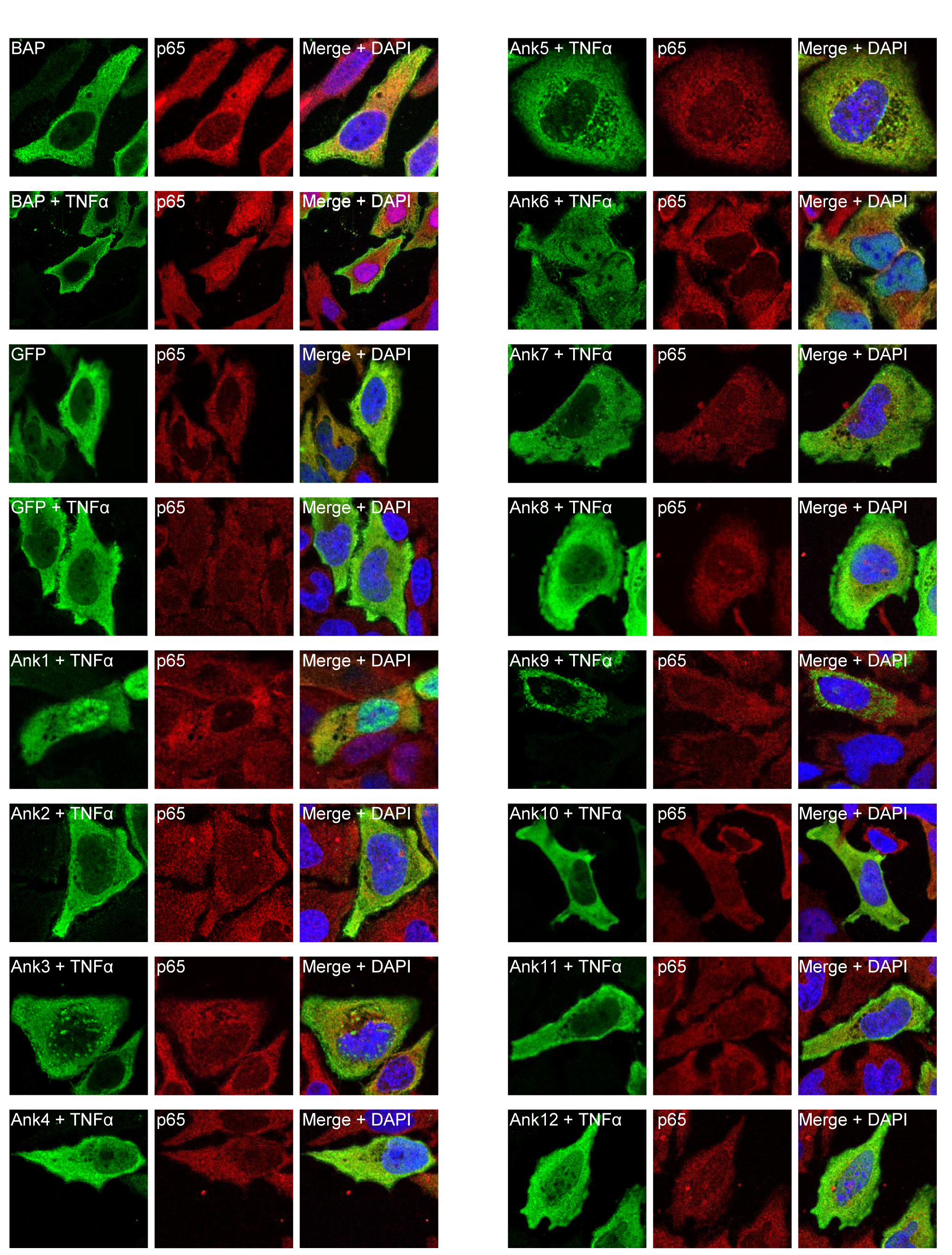

Supplement: S3 Fig — HeLa cells were transfected to express Flag-BAP; GFP; Flag-tagged Ank1, Ank2, Ank3, Ank4, Ank5, Ank6, Ank7, Ank8, Ank9, Ank11, Ank12; or GFP-Ank10. At 16 h, the cells were exposed to TNFα or vehicle control for 30 min, after which they were fixed, screened with antibodies specific for p65 and the Flag epitope or GFP and examined by confocal microscopy. Images of cells expressing a given recombinant Ank treated with vehicle control are not presented because in all such cases p65 was absent from the nucleus and looked exactly like the panels presented for BAP- or GFP-expressing cells not treated with TNFα. Results are representative of three independent experiments. (TIF) [file ppat.1007023.s003.tif]

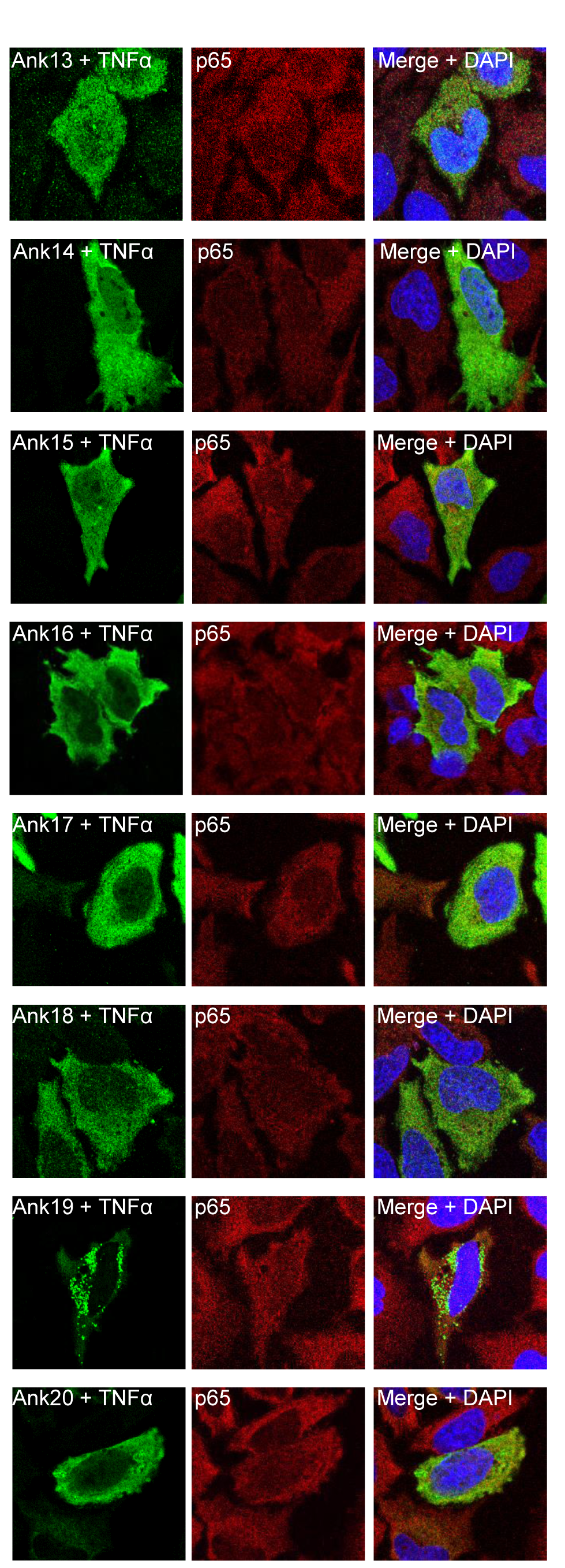

Supplement: S4 Fig — These data pair with data presented in S3 Fig. HeLa cells were transfected to express Flag-tagged Ank13, Ank15, Ank16, Ank17, Ank18, Ank19, Ank20, or GFP-Ank14. At 16 h, the cells were exposed to TNFα or vehicle control for 30 min, after which they were fixed, screened with antibodies specific for p65 and the Flag epitope or GFP and examined by confocal microscopy. Results are representative of three independent experiments. (TIF) [file ppat.1007023.s004.tif]

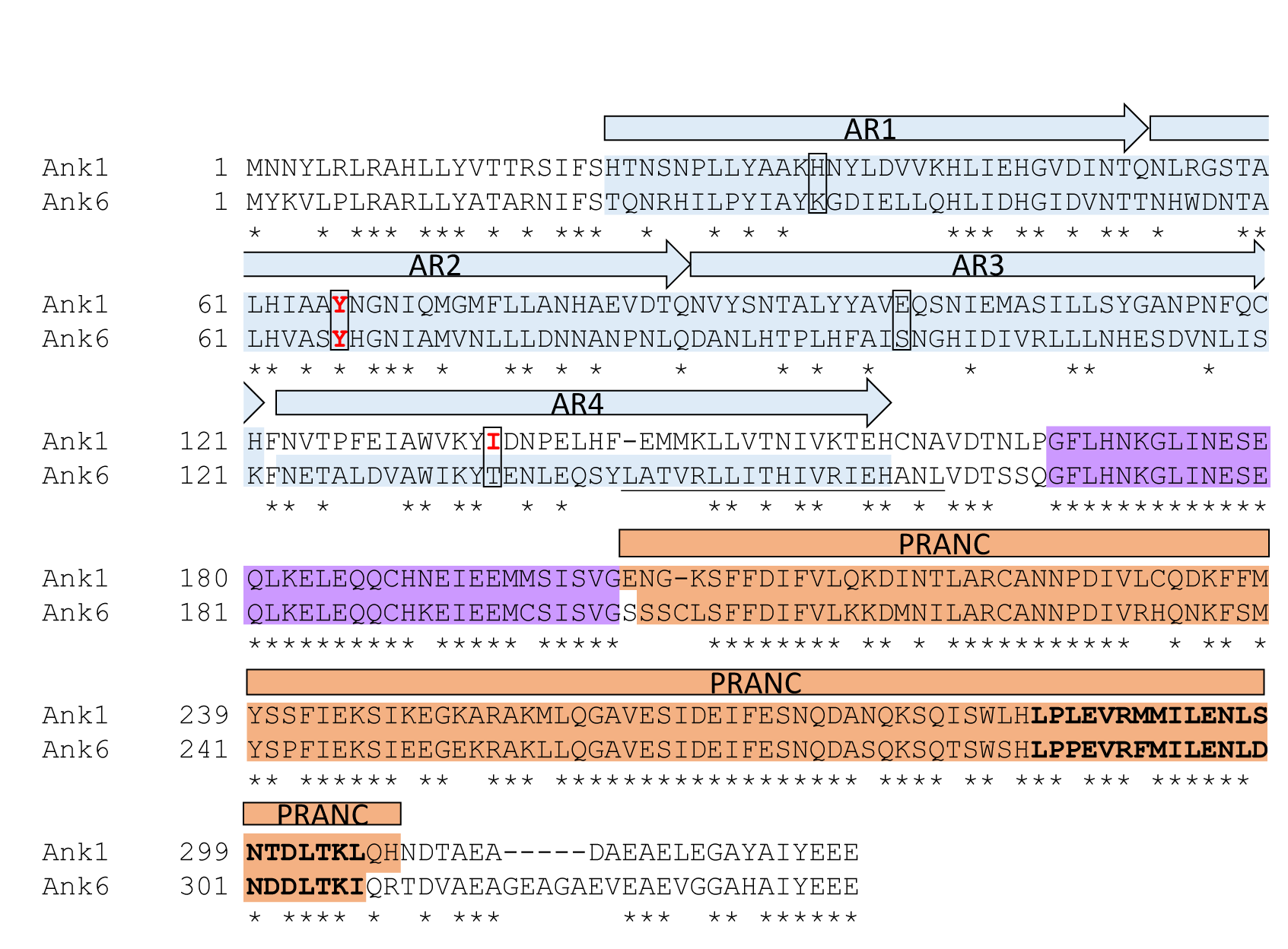

Supplement: S5 Fig — Amino acid positions are listed to the left of the sequences. The arrows labeled AR (ankyrin repeat) 1 through 4 correspond to the individual ankyrin repeats. The AR, ISR, and PRANC domains are shaded blue, purple, and orange, respectively. The non-shaded region of Ank1 (residues 122 to 157) that aligns with Ank6 AR4 (residues 122 to 158) bears 41.7% identity with AR4, but is not annotated as an AR. Because eukaryotic ankyrin repeat domains can facilitate nuclear localization through RanGDP binding at two consecutive ankyrin repeats provided that a hydrophobic residue is located at the thirteenth position in each of the two repeats, the thirteenth residue of each AR, including the potential fourth AR of Ank1, is boxed. Hydrophobic amino acids at this position are indicated by red boldface text. The putative NES of Ank6 at amino acids 143 to 161 is underlined. Residues that make up the F-box are denoted by black boldface text. Amino acids that are identical between Ank1 and Ank6 are underscored by asterisks. (TIF) [file ppat.1007023.s005.tif]

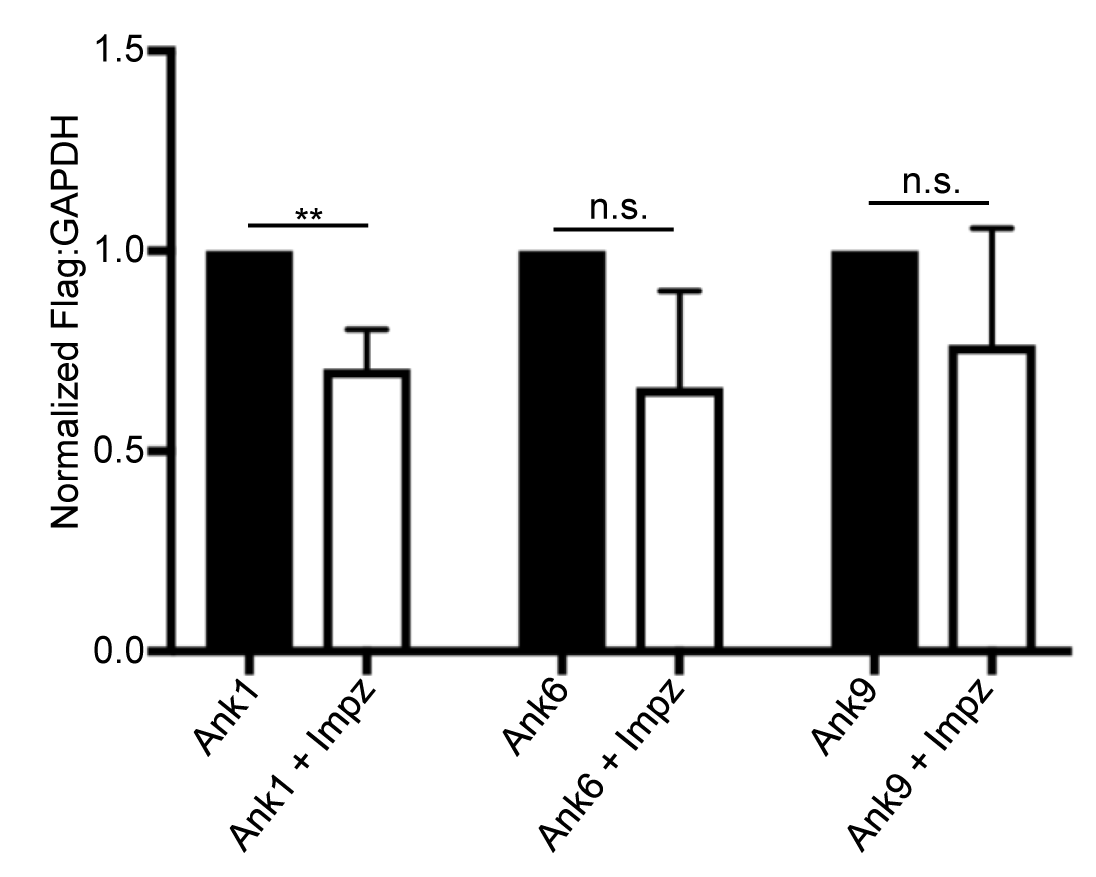

Supplement: S6 Fig — HeLa cells were transfected to express Flag-tagged Ank1, Ank6, or Ank9. At 16 h, the cells were treated with importazole or vehicle for 3 h. Following lysis and nuclear fractionation, Western blotted nuclear or cytosolic fractions were screened with antibodies against the Flag epitope, lamin A/C, and GAPDH. Mean ratios + SD of Flag:GAPDH densitometric signals from three separate Western blots, a representative image of which is presented in Fig 10A, were normalized to that for Flag-Ank9 to determine the effect of importazole on cytosolic levels of the ectopically expressed Flag-tagged Anks. Statistically significant (**P < 0.01) values are indicated. n.s., not significant. Data presented are representative of three independent experiments. (TIF) [file ppat.1007023.s006.tif]

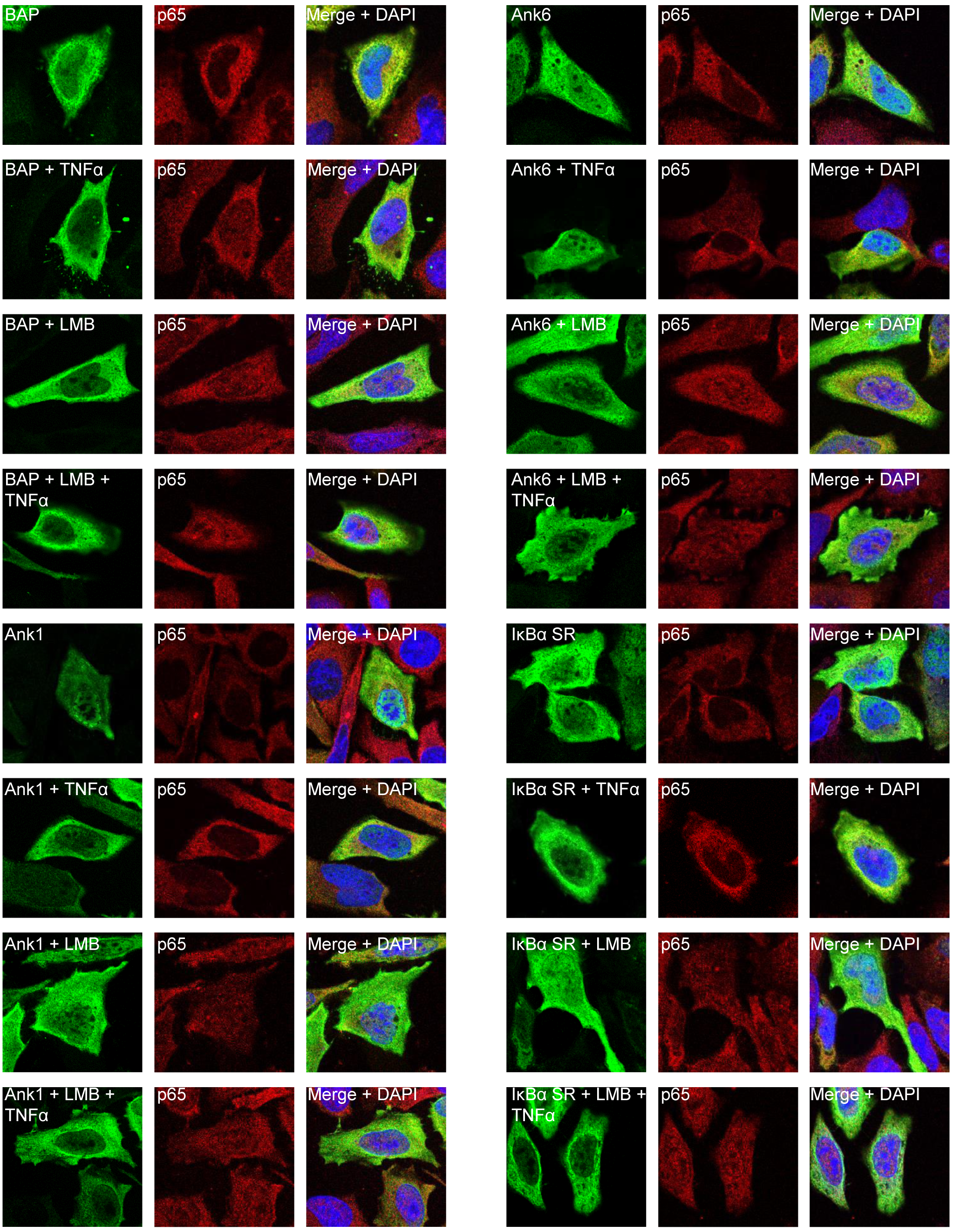

Supplement: S7 Fig — HeLa cells were transfected to express Flag tagged BAP, Ank1, Ank6, or IκBα SR. At 16 h, the cells were treated with LMB or vehicle control for 1 h. The media was replaced with media containing TNFα or vehicle for 30 min. The cells were then fixed, screened with antibodies specific for the Flag epitope and p65, and examined by confocal microscopy. Representative fluorescence images of cells viewed for Flag signal, p65, and merged images plus DAPI are presented. Results are representative of three independent experiments. (TIF) [file ppat.1007023.s007.tif]

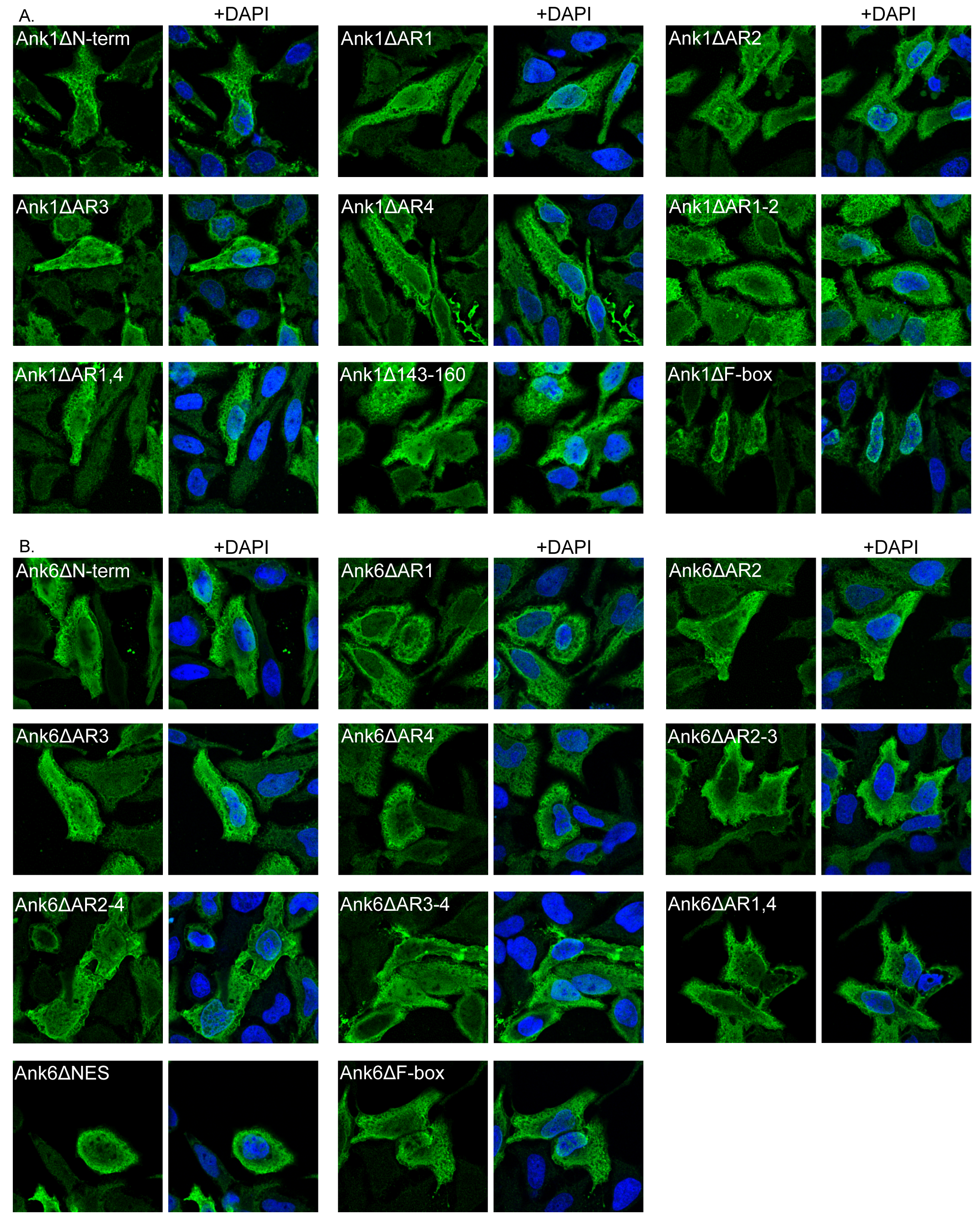

Supplement: S8 Fig — HeLa cells were transfected to express the indicated Flag-tagged deletion mutants of Ank1 or Ank6. At 16 h, the cells were fixed, screened with Flag tag antibody, stained with DAPI, and examined by confocal microscopy. Representative fluorescence images of cells viewed for Flag-tagged Ank1 (A) and Ank6 proteins (B) with and without DAPI are presented. Triplicate samples of 100 cells were counted per condition. Data presented are indicative of three experiments with similar results. (TIF) [file ppat.1007023.s008.tif]

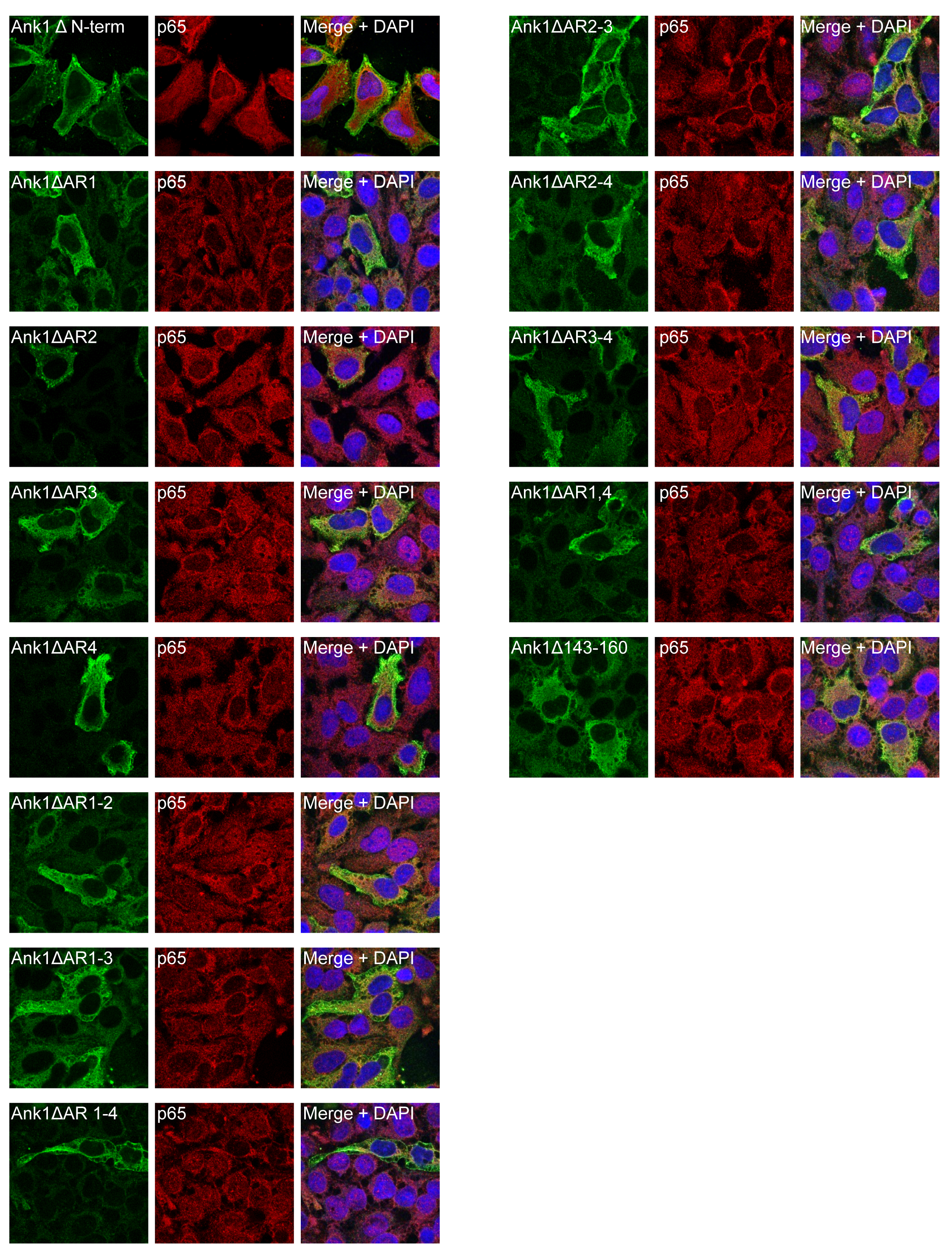

Supplement: S9 Fig — HeLa cells were transfected to express Flag-tagged BAP or the indicated Ank1 deletion mutants. At 16 h, the cells were exposed to TNFα for 30 min after which they were fixed, screened with antibodies specific for the Flag epitope and p65, and examined by confocal microscopy. Representative fluorescence images of cells viewed for Flag-tagged protein, p65, and merged images plus DAPI are presented. Data obtained for cells expressing Flag-tagged Ank1, Ank1△ISR, and Ank1△F-box, the latter two of which are compromised in the ability to inhibit p65 nuclear accumulation, are presented in Fig 17. (TIF) [file ppat.1007023.s009.tif]

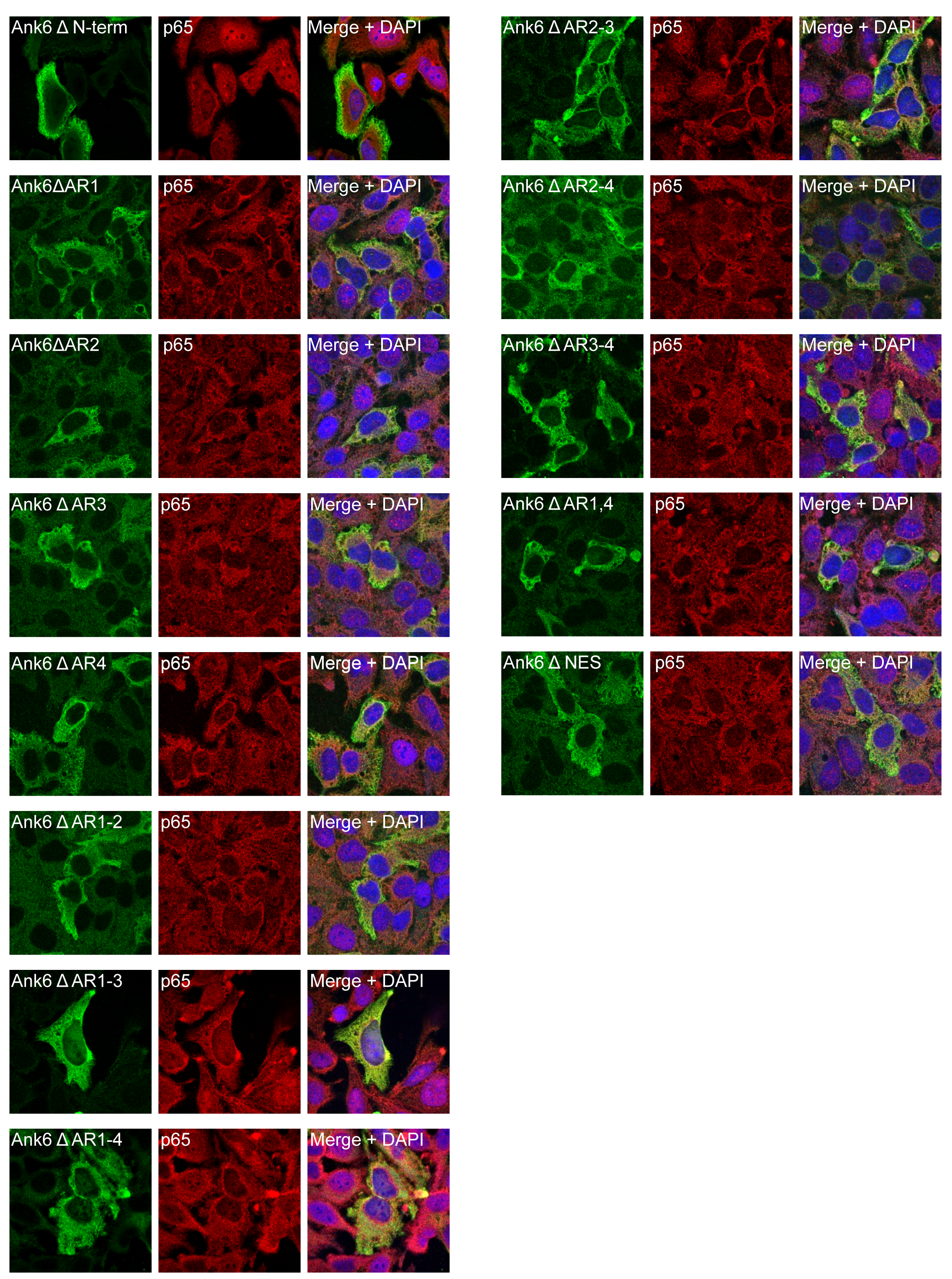

Supplement: S10 Fig — HeLa cells were transfected to express Flag-tagged BAP or the indicated Ank6 deletion mutant. At 16 h, the cells were exposed to TNFα for 30 min after which they either were fixed, screened with antibodies specific for the Flag epitope and p65, and examined by confocal microscopy. Representative fluorescence images of cells viewed for Flag-tagged protein, p65, and merged images plus DAPI are presented. Data obtained for cells expressing Flag-tagged Ank6, Ank6△ISR, and Ank6△F-box, the latter two of which are compromised in the ability to inhibit p65 nuclear accumulation, are presented in Fig 17. (TIF) [file ppat.1007023.s010.tif]
