# Supplementary material for: Orientia tsutsugamushi uses two Ank effectors to modulate NF-κB p65 nuclear transport and inhibit NF-κB transcriptional activation
Source: PLoS Pathog. 2018 May 7;14(5):e1007023. doi: 10.1371/journal.ppat.1007023 (PMC5957444; doi:10.1371/journal.ppat.1007023)
Supplement: S1 Table — (PDF) [file ppat.1007023.s011.pdf]

S1 Table. Amino acid identities between regions of *O. tsutsugamushi* str. Ikeda Ank1 and Ank6

Region of interest	Amino acid positions	Percent identity
Whole sequence	Ank1: 1-329<br>Ank6: 1-336	58.6%
N-terminus	Ank1: 1-21<br>Ank6: 1-21	61.9%
Ankyrin repeat domain	Ank1: 22-157[a]<br>Ank6: 22-158	37.2%
Intervening sequence region (ISR) between the ankyrin repeat domain and PRANC domain	Ank1: 167-201<br>Ank6: 168-202	84.1%
PRANC domain	Ank1: 202-307<br>Ank6: 204-309	76.9%
F-box	Ank1: 285-305<br>Ank6: 287-307	76.2%
C-terminus	Ank1: 308-329<br>Ank6: 310-336	55.6%

[a]Includes the region of Ank1 that aligns with and exhibits 41.7% identity with ankyrin repeat four of Ank6, but was not annotated as an ankyrin repeat in the original Genbank entry.
