# Supplementary material for: Orientia tsutsugamushi uses two Ank effectors to modulate NF-κB p65 nuclear transport and inhibit NF-κB transcriptional activation
Source: PLoS Pathog. 2018 May 7;14(5):e1007023. doi: 10.1371/journal.ppat.1007023 (PMC5957444; doi:10.1371/journal.ppat.1007023)
Supplement: S2 Table — (PDF) [file ppat.1007023.s012.pdf]

S2 Table. Correlation of Flag-Ank1 and Flag-Ank6 abilities to interact with importin β1 and translocate into the nucleus

Flag-tagged protein	Importin β1 interaction[a]	Nuclear translocation phenotype[b]
Ank1	++++	Nuclear translocation
Ank1ΔN-terminus	+++++	Nuclear translocation
Ank1ΔAR1	++++	Nuclear translocation
Ank1ΔAR2	+++	Nuclear translocation
Ank1ΔAR3	++	Nuclear translocation
Ank1ΔAR4	++	Nuclear translocation
Ank1ΔAR1-2	++	Nuclear translocation
Ank1ΔAR1-3	+++	Reduced nuclear translocation; concentrated around periphery of nucleus
Ank1ΔAR1-4	+	Reduced nuclear translocation; concentrated around periphery of nucleus
Ank1ΔAR2-3	+	Reduced nuclear translocation; concentrated around periphery of nucleus
Ank1ΔAR2-4	+	Reduced nuclear translocation; concentrated around periphery of nucleus
Ank1ΔAR3-4	++	Reduced nuclear translocation; concentrated around periphery of nucleus
Ank1ΔAR1,4	++	Nuclear translocation
Ank1Δ143-160	++	Nuclear translocation
Ank1ΔISR	++	Nuclear translocation
Ank1ΔF-box	++++	Nuclear translocation
Ank6	++++	Nuclear translocation
Ank6ΔN-terminus	++++	Nuclear translocation
Ank6ΔAR1	+++	Reduced nuclear translocation
Ank6ΔAR2	++	Reduced nuclear translocation
Ank6ΔAR3	++++	Nuclear translocation
Ank6ΔAR4	+++	Reduced nuclear translocation
Ank6ΔAR1-2	++	Reduced nuclear translocation; concentrated around periphery of nucleus
Ank6ΔAR1-3	+	Reduced nuclear translocation; concentrated around periphery of nucleus
Ank6ΔAR1-4	++	Reduced nuclear translocation; concentrated around periphery of nucleus
Ank6ΔAR2-3	++	Reduced nuclear translocation
Ank6ΔAR2-4	++	Reduced nuclear translocation
Ank6ΔAR3-4	+++	Reduced nuclear translocation
Ank6ΔAR1,4	++	Reduced nuclear translocation
Ank6ΔNES	++	Reduced nuclear translocation
Ank6ΔISR	+++	Reduced nuclear translocation
Ank6ΔF-box	+++++	Nuclear translocation

[a]Scoring based on densitometry values presented in Fig. 15B.
[b]Phenotype based on results presented in Fig. 16 and S8 Fig.
