# Supplementary material for: Orientia tsutsugamushi uses two Ank effectors to modulate NF-κB p65 nuclear transport and inhibit NF-κB transcriptional activation
Source: PLoS Pathog. 2018 May 7;14(5):e1007023. doi: 10.1371/journal.ppat.1007023 (PMC5957444; doi:10.1371/journal.ppat.1007023)
Supplement: S3 Table — (PDF) [file ppat.1007023.s013.pdf]

S3 Table. Oligonucleotide primers used in this study

Primer Designation[a]	Sequence (5' to 3')
pFLAG-Ank1-1F	TTGCGGCCGCGAATTCCATGAACAACTACCTGAGAC
pFLAG-Ank1-990R	ATGCCACCCGGGATCCTCACTCTTCCTCGTAGATGGC
pFLAG-Ank1-52F	TTGCGGCCGCGAATTCCTCCATCTTCAGCCACACCAAC
pFLAG-Ank1-990R-EcoRI	ATCTATCGATGAATTCTCACTCTTCCTCGTAGATGGCGTAGGC
Ank1-160-Ank1-66R	**GCTCCCCCTCAGGTT**GTGGCTGAAGATGGACCTG
Ank1-160F	AACCTGAGGGGGAGCACA
pFLAG-Ank1pBMC-1F	TTGCGGCCGCGAATTCCATGAACAAY[b]TAYCTGAGGCTS[c]AGG
Ank1-472-Ank1-366R	**GTCCACGGCGTTGCA**GAAGTGACATTGGAAGTTGG
Ank1-472F	TGCAACGCCGTGGACACA
Ank1-265-Ank1-66R	**GGCGGTGTTGCTGTA**GTGGCTGAAGATGGACCT
Ank1-265F	TACAGCAACACCGCCCTG
Ank1-364-Ank1-66R	**GGGGGTCACGTTGAA**GTGGCTGAAGATGGACCT
Ank1-364F	TTCAACGTGACCCCCTTTG
Ank1-472-Ank1-66R	**GTCCACGGCGTTGCA**GTGGCTGAAGATGGACCT
Ank1-472-Ank1-165R	**GTCCACGGCGTTGCA**CAGGTTCTGAGTATTGATGT
Ank1-472-Ank1-264R	**GTCCACGGCGTTGCA**CACGTTCTGGGTGTCCAC
Ank1-166-Ank1-66R	**AGCTGTGCTCCCCCT**GTGGCTGAAGATGGACCT
Ank1-166F	AGGGGGAGCACAGCTCTG
Ank1-481-Ank1-426R	**CAGGTTTGTGTCCAC**GAAGTGCAGCTCGGGGTT
Ank1-481F	GTGGACACAAACCTGCCC
Ank1-604-Ank1-498R	**GGACTTGCCGTTCTC**GGGCAGGTTTGTGTCCAC
Ank1-604F	GAGAACGGCAAGTCCTTC
pFLAG-Ank1-4F[d]	ATCGGAATTCAAACAACTACCTGAGACTGAGG
pFLAG-Ank1-852R[d]	CGATGTCGACTTAGTGCAGCCAGCTGATCTGG
pFLAG-Ank6-1F	TTGCGGCCGCGAATTCCATGTACAAGGTTCTGCCA
pFLAG-Ank6-1011R	ATGCCACCCGGGATCCTCACTCCTCTTCGTATATTGCG
pFLAG-Ank6-52F	TTGCGGCCGCGAATTCCAACATTTTTAGCACCCAAAATCGACACATAC
pFLAG-Ank6-1011R-EcoRI	ATCTATCGATGAATTCTCACTCCTCTTCGTATATTGCGTG
pFLAG-Ank6-160F	AACCACTGGGACAACACG
Ank6-160-Ank6-66R	**GTTGTCCCAGTGGTT**GGTGCTAAAAATGTTCCTTGCC
pFLAG-Ank6pBMC-1F	TTGCGGCCGCGAATTCCATGTACAAGGTSCTSCCYCTG
Ank6-475-Ank6-366R	**GTCCACCAGGTTGGC**AAACTTACTGATGAGGTTGAC
Ank6-475F	GCCAACCTGGTGGACACC
Ank6-265-Ank6-66R	**CGGGGTGTGAAGGTT**GGTGCTAAAAATGTTCCTTG
Ank6-265F	AACCTTCACACCCCGCTG
Ank6-364-Ank6-66R	**GGCGGTCTCGTTAAA**GGTGCTAAAAATGTTCCTTG
Ank6-364F	TTTAACGAGACCGCCCTTG
Ank6-475-Ank6-66R	**GTCCACCAGGTTGGC**GGTGCTAAAAATGTTCCTTG
Ank6-475-Ank6-165R	**GTCCACCAGGTTGGC**GTGGTTGGTTGTATTAACGT
Ank6-475-Ank6-264R	**GTCCACCAGGTTGGC**CGCGTCCTGCAGATTGGG
Ank6-166-Ank6-66R	**TGCCGTGTTGTCCCA**GGTGCTAAAAATGTTCCTTG

Ank6-166F	TGGGACAACACGGCACTG
Ank6-484-Ank6-426R	**GCTTGAGGTGTCCAC**ATAGCTCTGTTCCAGGTTC
Ank6-484F	GTGGACACCTCAAGCCAG
Ank6-607-Ank6-501R	**GAGGCAGCTGGAGGA**CTGGCTTGAGGTGTCCAC
Ank6-607F	TCCTCCAGCTGCCTCAGC
pFLAG-Ank6-4F[d]	<u>GAATCGGAATTCA</u>TACAAGGTTCTGCCACTCAG
pFLAG-Ank6-858R[d]	<u>CGATGTCGAC</u>TTAGTGGGACCAACTGGTTTGG

[a]F and R refer to primers that bind to the sense and antisense strand, respectively. The number immediately preceding the F or R denotes the first nucleotide position where the primer binds. Underlined nucleotides correspond to p3XFLAG-CMV-7.1 vector sequence. Unformatted text corresponds to *ank* gene nucleotides. Boldface nucleotides correspond to a 15-nucleotide stretch within the targeted *ank* gene that serves as a homology site for InFusion ligation to occur.

[b]Degenerate primer where Y represents C or T.

[c]Degenerate primer where S represents C or G.

[d]Ligation independent cloning primers were used to generate constructs encoding Ank1 or Ank6 lacking the C-terminal F-box as described in reference [25].
