# Supplementary material for: Orientia tsutsugamushi uses two Ank effectors to modulate NF-κB p65 nuclear transport and inhibit NF-κB transcriptional activation
Source: PLoS Pathog. 2018 May 7;14(5):e1007023. doi: 10.1371/journal.ppat.1007023 (PMC5957444; doi:10.1371/journal.ppat.1007023)
Supplement: S4 Table — (PDF) [file ppat.1007023.s014.pdf]

S4 Table. Primers utilized for InFusion generation of constructs encoding truncated Anks

Construct name	Primer Sets Utilized
pFLAG-Ank1ΔN-terminus	pFLAG-Ank1-52F and pFLAG-990R-EcoRI
pFLAG-Ank1ΔAR1	pFLAG-Ank1-1F and Ank1-160-Ank1-66R<br>Ank1-160F and pFLAG-Ank1-990R
pFLAG-Ank1ΔAR2	pFLAG-Ank1pBMC-1F[a] and pFLAG-Ank1-990R[a]
pFLAG-Ank1ΔAR3	pFLAG-Ank1pBMC-1F[b] and pFLAG-Ank1-990R[b]
pFLAG-Ank1ΔAR4	pFLAG-Ank1-1F and Ank1-472-Ank1-366R<br>Ank1-472F and pFLAG-Ank1-990R
pFLAG-Ank1ΔAR1-2	pFLAG-Ank1-1F and Ank1-265-Ank1-66R<br>Ank1-265F and pFLAG-Ank1-990R
pFLAG-Ank1ΔAR1-3	pFLAG-Ank1-1F and Ank1-364-Ank1-66R<br>Ank1-364F and pFLAG-Ank1-990R
pFLAG-Ank1ΔAR1-4	pFLAG-Ank1-1F and Ank1-472-Ank1-66R<br>Ank1-472F and pFLAG-Ank1-990R
pFLAG-Ank1ΔAR2-3	pFLAG-Ank1pBMC-1F[c] and pFLAG-Ank1-990R[c]
pFLAG-Ank1ΔAR2-4	pFLAG-Ank1-1F and Ank1-472-Ank1-165R<br>Ank1-472F and pFLAG-Ank1-990R
pFLAG-Ank1ΔAR3-4	pFLAG-Ank1-1F and Ank1-472-Ank1-264R<br>Ank1-472F and pFLAG-Ank1-990R
pFLAG-Ank1ΔAR1,4	pFLAG-Ank1-1F and Ank1-166-Ank1-66R<br>Ank1-166F[d] and pFLAG-Ank1-990R[d]
pFLAG-Ank1ΔNES	pFLAG-Ank1-1F and Ank1-481-Ank1-426R<br>Ank1-481F and pFLAG-Ank1-990R
pFLAG-Ank1ΔISR	pFLAG-Ank1-1F and Ank1-604-Ank1-498R<br>Ank1-604F and pFLAG-Ank1-990R
pFLAG-Ank1ΔF-box[e]	pFLAG-Ank1-4F and pFLAG-Ank1-852R
pFLAG-Ank6ΔN-terminus	pFLAG-Ank6-52F and pFLAG-Ank6-1011R-EcoRI
pFLAG-Ank6ΔAR1	pFLAG-Ank6-1F and Ank6-160-Ank6-66R<br>Ank6-160F and pFLAG-Ank6-1011R
pFLAG-Ank6ΔAR2	pFLAG-Ank6pBMC-1F[f] and pFLAG-Ank6-1011R[f]
pFLAG-Ank6ΔAR3	pFLAG-Ank6pBMC-1F[g] and pFLAG-Ank6-1011R[g]
pFLAG-Ank6ΔAR4	pFLAG-Ank6-1F and Ank6-475-Ank6-366R<br>Ank6-475F and pFLAG-Ank6-1011R
pFLAG-Ank6ΔAR1-2	pFLAG-Ank6-1F and Ank6-265-Ank6-66R<br>Ank6-265F and pFLAG-Ank6-1011R
pFLAG-Ank6ΔAR1-3	pFLAG-Ank6-1F and Ank6-364-Ank6-66R<br>Ank6-364F and pFLAG-Ank6-1011R
pFLAG-Ank6ΔAR1-4	pFLAG-Ank6-1F and Ank6-475-Ank6-66R<br>Ank6-475F and pFLAG-Ank6-1011R
pFLAG-Ank6ΔAR2-3	pFLAG-Ank6pBMC-1F[h] and pFLAG-Ank6-1011R[h]
pFLAG-Ank6ΔAR2-4	pFLAG-Ank6-1F and Ank6-475-Ank6-165R<br>Ank6-475F and pFLAG-Ank6-1011R
pFLAG-Ank6ΔAR3-4	pFLAG-Ank6-1F and Ank6-475-Ank6-264R<br>Ank6-475F and pFLAG-Ank6-1011R
pFLAG-Ank6ΔAR1,4	pFLAG-Ank6-1F and Ank6-166-Ank6-66R

	Ank6-166F[i] and pFLAG-Ank6-1011R[i]
pFLAG-Ank6ΔNES	pFLAG-Ank6-1F and Ank6-484-Ank6-426R<br>Ank6-484F and pFLAG-Ank6-1011R
pFLAG-Ank6ΔISR	pFLAG-Ank6-1F and Ank6-607-Ank-501R<br>Ank6-607F and pFLAG-Ank6-1011R
pFLAG-Ank6ΔF-box[e]	pFLAG-Ank6-4F and pFLAG-Ank6-858R

[a]DNA source for PCR amplification was pBMC-Ank1ΔAR2.
[b]DNA source for PCR amplification was pBMC-Ank1ΔAR3.
[c]DNA source for PCR amplification was pBMC-Ank1ΔAR2-3.
[d]DNA source for PCR amplification was pBMC-Ank1ΔAR4.
[e]This construct was generated by ligation independent cloning.
[f]DNA source for PCR amplification was pBMC-Ank6ΔAR2.
[g]DNA source for PCR amplification was pBMC-Ank6ΔAR3.
[h]DNA source for PCR amplification was pBMC-Ank6ΔAR2-3.
[i]DNA source for PCR amplification was pBMC-Ank6ΔAR4.
